# Epigenome screening highlights that JMJD6 confers an epigenetic vulnerability and mediates sunitinib sensitivity in renal cell carcinoma

**DOI:** 10.1002/ctm2.328

**Published:** 2021-02-14

**Authors:** Chuanjie Zhang, Xuan Lu, Jingyi Huang, Hongchao He, Li Chen, Yihan Liu, Haofei Wang, Yang Xu, Siwei Xing, Xiaohao Ruan, Xiaoqun Yang, Lu Chen, Danfeng Xu

**Affiliations:** ^1^ Department of Urology Ruijin Hospital Shanghai Jiao Tong University School of Medicine Shanghai China; ^2^ Department of Pharmacology Basic Medical College Anhui Medical University Hefei China; ^3^ Department of Pharmacy Shanghai Xuhui District Central Hospital Xuhui Hospital of Zhongshan Hospital Affiliated to Fudan University Shanghai China; ^4^ Department of Epidemiology and Biostatistics School of Public Health Nanjing Medical University Nanjing China; ^5^ Department of Pathology Ruijin Hospital Shanghai Jiao Tong University School of Medicine Shanghai China

**Keywords:** epigenetic vulnerability, JMJD6, sunitinib, super‐enhancers

## Abstract

Aberrant epigenetic reprogramming represents a hallmark of renal cell carcinoma (RCC) tumorigenesis and progression. Whether there existed other epigenetic vulnerabilities that could serve as therapeutic targets remained unclear and promising. Here, we combined the clustered regularly interspaced short palindromic repeats functional screening results and multiple RCC datasets to identify JMJD6 as the potent target in RCC. JMJD6 expression correlated with poor survival outcomes of RCC patients and promoted RCC progression in vitro and in vivo. Mechanistically, aberrant p300 led to high JMJD6 expression, which activated a series of oncogenic crosstalk. Particularly, high‐throughput sequencing data revealed that JMJD6 could assemble super‐enhancers to drive a list of identity genes in kidney cancer, including VEGFA, β‐catenin, and SRC. Moreover, this JMJD6‐mediated oncogenic effect could be suppressed by a novel JMJD6 inhibitor (SKLB325), which was further demonstrated in RCC cells, patient‐derived organoid models, and in vivo. Given the probable overlapped crosstalk between JMJD6 signature and tyrosine kinase inhibitors downstream targets, targeting JMJD6 sensitized RCC to sunitinib and was synergistic when they were combined together. Collectively, this study indicated that targeting JMJD6 was an effective approach to treat RCC patients.

## Graphical abstract

The *CRISPR/Cas9* functional screening data and multiple RCC datasets were used to identify JMJD6 as the epigenetic vulnerability in RCC. Accumulated JMJD6 mainly constitutes super‐enhancers to alter oncogenic crosstalk and targeting JMJD6 was an effective approach to suppress RCC progression.

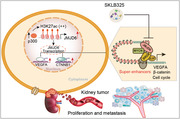

**Abbreviations**
ChIP: chromatin immunoprecipitation
DEG: differentially expressed gene
GeCK: genome‐wide clustered regularly interspaced short palindromic repeats (CRISPR)‐associated nuclease Cas9 genome editing system knockout screen
GO: Gene Ontology
IHC: immunohistochemistry
ICGC, International Cancer Genome Consortium: International Cancer Genome Consortium
JMJD6: Jumonji domain‐containing 6
OS: overall survival
PDX: patient‐derived xenograft
qPCR: quantitative polymerase chain reaction
RCC: renal cell carcinoma
ROSE: the rank oriented of super enhances
RTK: receptor tyrosine kinase
SE: super‐enhancers
SR: sunitinib‐resistant
SRC: SRC proto‐oncogene, nonreceptor tyrosine kinase
TCGA: The Cancer Genome Atlas
VEGFA: vascular endothelial growth factor A

## 1 INTRODUCTION

Kidney cancer is a common type of urinary malignancy with a relatively poor prognosis and an incidence rate that has increased in recent years.^1^ According to 2020 cancer statistics, the estimated number of new kidney cancer cases in the United States may reach 73,750 and result in approximately 14,830 deaths.^2^ Kidney cancer is a heterogeneous disease that includes several histological subtypes, and the most common pathological subtype is clear cell renal cell carcinoma (ccRCC).^3^ Recently, high‐throughput sequencing data and etiology analysis revealed that ccRCC harbors well‐known mutations in a series of chromatin modifier genes, such as *VHL*, *PBRM1*, *SETD2*, and *KDM5C*.^4^, ^5^ Aberrant epigenetic programming has been recognized as a hallmark of renal cell carcinoma (RCC) tumorigenesis and progression.^6^ Targeting epigenetic modifiers have exhibited satisfactory effects, such as JQ1, HDAC inhibitors, or EZH2 inhibitors. As previously reported, JQ1, one bromodomain BET inhibitor, was proved effectively to suppress tumor growth via specially targeting BRD4.^7^, ^8^ Besides, EZH2 inhibition via Tazemetostat was proven to be an effective strategy for the suppressing H3K27M‐mutant pediatric gliomas or lymphoma.^9^, ^10^ As a result, screening and developing novel epigenetic inhibitors for use in RCC are worthy and promising endeavors.^11^, ^12^ However, in recent years, therapeutics for advanced RCC have been limited to mainly antiangiogenic targeted therapies and immunotherapies.^13^ Furthermore, only a portion of patients benefit from long‐term drug responses that suppress tumors.^14^ Sunitinib belongs to a multi‐targeted receptor tyrosine kinase (RTK) inhibitor, which is a first‐in‐line small‐molecule drug for advanced RCC.^15^, ^16^ However, the general treatment efficacy of sunitinib or PDL1 remains limited owing to the frequent development of resistance, and more than 30% of RCC cases had progressed into the terminal stage at the time of diagnosis.^17^, ^18^, ^19^ Thus, there is urgent demand for discovering novel insights and therapeutic strategies for RCC, especially those targeting abnormal epigenetic drivers.

Currently, the genome‐wide clustered regularly interspaced short palindromic repeats (CRISPR)‐associated nuclease Cas9 genome editing system knockout screen (GeCK) has emerged as an effective next‐generation approach for functional screening.^20^, ^21^ The loss‐of‐function genetic screens are mainly focused on the potential fitness of candidates that mediate tumor growth, drug responses, cancer metastasis, and drug resistance.^22^ In addition, researchers have released comprehensive screening results from 33 tumor cell lines including RCC, providing useful resources for the prioritization of cancer epigenetic targets in our study.^23^ Furthermore, countless high‐throughput sequencing data obtained during cancer discovery are produced daily, and cloud computing algorithms have been rapidly developed. The Pan‐cancer Analysis of Whole Genomes (PCAWG) consortium was established for investigating oncogenic drivers based on The Cancer Genome Atlas (TCGA) and International Cancer Genome Consortium (ICGC) resources, which has been a research hotpot.^24^, ^25^ As a result, we combined the two powerful technologies incorporating GeCK screening results and sequencing data from TCGA/ICGC cohorts to identify robust epigenetic vulnerability in RCC.

The Jumonji domain‐containing 6 (JMJD6) gene, belonging to a family of JmjC‐domain‐containing proteins, was identified as an iron (Fe^2+^)‐ and 2‐oxoglutarate‐dependent dioxygenase.^26^, ^27^ As reported, JMJD6 can alter downstream gene expression levels by interacting with a coactivator of BRD4 and demethylating histone H4 at arginine 3, resulting in abnormal distal promoter proximal pausing of Pol II release.^28^, ^29^ JMJD6 is a decisive regulator of the rapid physiological responses to estrogen depending on its demethylase activity.^30^ In addition, JMJD6 can also function as a lysyl‐hydroxylase to constitute complexes that lead to p53 hydroxylation and inactivation.^31^ Meanwhile, JMJD6 was reported to downregulate H4K16ac independently of the enzymatic activity that modulates the epigenome around DNA lesions.^32^ Previous studies have suggested that JMJD6 can regulate multiple biological processes, including the cell cycle, proliferation, and tumorigenesis.^33^, ^34^ Knockouts of JMJD6 could lead to severe defects in mice, which suggested critical roles of JMJD6 in development.^35^ Furthermore, JMJD6 could accompany with BRD4 to regulate the activity of CDK9 and RNA polymerase II complex.^36^ However, the specific function and therapeutic significance of JMJD6 in RCC are indefinite. Given the potential value of JMJD6 in cancer treatment, researchers have accordingly designed an inhibitor, SKLB325, based on the crystal structure of the jmjC domain of JMJD6, which was demonstrated to possess remarkable antitumor effects in ovarian cancer.^33^ Whether the JMJD6 inhibitor SKLB325 can effectively suppress RCC growth and become a novel alternative drug is essential to figure out.

In the current study, we combined GeCK screening data and RCC cohorts to identify JMJD6 as a pivotal chromatin modifier in RCC. Overexpressed JMJD6 could promote RCC progression in vitro and in vivo through remodeling the oncogenic transcriptome profiles. We proposed that JMJD6 may be a promising predictor of prognosis and therapeutic target for treating RCC.

## 2 MATERIALS AND METHODS

### 2.1 Cell culture and RCC patient samples

The 293T cells and kidney cancer cell lines (786‐O, ACHN, and Caki‐1) were obtained from the American Type Culture Collection (ATCC). 786‐O, ACHN, 769‐P Caki‐1, and A498 belong to human renal clear cell adenocarcinoma cells, all originating from renal tubular epithelial cells. The RCC cell lines were cultured in medium containing RPMI‐1640 (Thermo Fisher Scientific, Waltham, MA, USA) supplemented with 10% FBS and 1% penicillin/streptomycin (Invitrogen, Carlsbad, CA, USA), whereas the 293T cells were maintained in DMEM medium with 10% FBS. All cells were kept at 37°C under 5% CO_2_. The cells were transiently transfected with plasmids or siRNAs using Lipofectamine 3000 (Thermo, USA). The resected RCC tumors and matched normal tissues were obtained from the Department of Urinary Surgery, Ruijin Hospital, Shanghai Jiaotong University School of Medicine, 197 Ruijin Second Road, Shanghai, 200025, China. All samples were collected according to the protocols approved by the institutional review boards, and informed consent was obtained from all subjects before samples collection. All resected RCC tumors were confirmed by two pathologists.

1 **HIGHLIGHTS**

- *CRISPR/Cas9* screen data and RCC cohorts identify JMJD6 as a novel epigenetic vulnerability in RCC.
- P300‐mediated activation of JMJD6 constitutes super‐enhancers to drive canonical kidney cancer crosstalk.
- SKLB325 is effective to treat RCC and synergistic with sunitinib.

### 2.2 Lentiviral infection, stable cell generation, and siRNA knock‐down assay

We purchased the pLKO.3G GFP‐shRNA plasmids from Addgene. After 48 h of transfection, the virus supernatant was obtained through centrifugation. We infected the cells with collected viruses combined with polybrene for 48 h, which were sorted by GFP signals. After seeded in the 24‐well plates, the kidney cancer cell lines (786‐O and ACHN) were then transfected with 20 nM siRNA oligos via RNAiMax reagent (Life Technology). Western blot assays were used to confirm the knockdown efficiency. The specific sequences of siRNA targets are summarized in Table S1.

### 2.3 Western blot assays

Lysis buffer with protease inhibitors was used to extract total proteins, which were separated by 8% SDS‐PAGE gels. Then, the proteins were transferred onto PVDF membranes (Millipore, Bedford, MA). Moreover, the membranes were blocked with 5% nonfat milk and incubated with specific primary antibodies overnight. After washing three times in TBS containing 0.1% Tween‐20, the membranes were incubated with the secondary antibody for 1 h at 4°C temperature. The ECL chemiluminescence system (Santa Cruz Biotechnology) was used to visualize the protein bands. The information of antibody used in the study is summarized and shown in Table S1.

### 2.4 Cell proliferation analysis, colony formation assays, and migration assay

The Cell Counting Kit‐8 (CCK‐8) was utilized to calculate the cell proliferation rate according to the manufacturer's protocol (Dojindo Laboratories, Japan). First, with a density of 1000 cells per well, the cells were seeded onto the 96‐well plates. From Day 2 to Day 8, 10 μl of the CCK‐8 solution was put into the cell culture. The microplate absorbance reader (Bio‐Rad) was used to detect the resulting color at 450 nm. Each experiment was repeated in triplicate. With a density of 1 × 10^3^ individual cells per well, the RCC cell lines were seeded onto six‐well plates in triplicate. Followed by staining with Giemsa dye for 20 min (Solarbio, China), the cell lines were fixed with 100% methanol for 5 min. Trans‐well (Costar) migration and invasion assays were conducted to assess the cell migration and invasion abilities. For migration assay, the lower chamber was filled with DMEM mixed with 10% FBS and the upper chamber was covered by 3 × 10^4^ cells. After 48 h, a cotton swab was used to remove the nonmigrating cells on the upper chambers, whereas the migrated cells below the filter were stained and calculated in eight different positions. Transwell inserts (Costar) coated with Matrigel (BD Biosciences)/fibronectin (BD Biosciences) was utilized to perform the Matrigel invasion assays.

### 2.5 Single‐cell JMJD6‐knockout clone generation

Guide oligos targeting JMJD6 were cloned in pX459 plasmid. 786‐O cells were cultured and transfected with pX459 constructs for 2 days. Then, 1 μg/ml puromycin was added into the medium to screen the remaining cells. The monoclonal cell line was isolated and obtained by seeding living cells onto 96‐well plate. Western blot assay was used to confirm the knockout efficiency of cells along with validations by Sanger sequencing method. The specific sequences of sgRNAs in the current study are summarized in Table S1.

### 2.6 Chromatin immunoprecipitation sequencing and data analysis, and chromatin immunoprecipitation coupled with quantitative real‐time polymerase chain reaction

Total RNA extracted from the indicated cells was subjected to HiSeq RNA‐Seq, which was performed by BGI Tech Solutions Co. Each sample contained pooled RNA from three biological replicas and was mixed with an equal mass of RNA to minimize variation across samples. Transcriptome reads from RNA‐Seq experiments were mapped to the reference genome (hg19) using the Bowtie tool. The gene expression level was quantified by the RSEM software package and the DEGs were detected with the Poisson distribution method. For chromatin immunoprecipitation (ChIP) sequencing (ChIP‐seq) analysis, formaldehyde (1%, 12 min)‐fixed cells were sheared to achieve chromatin fragmented to a range of 200–700 bp using an Active Motif EpiShear Probe Sonicator, after which ChIP‐Seq assays were performed by Active Motif Inc. Two ChIP coupled with quantitative polymerase chain reaction (ChIP‐qPCR) primer pairs that overlap the p300 binding site of the human JMJD6 promoter region are designed as in Table S1. Samples were run in triplicate, and data from p300 IP or control IP were calculated as enrichment relative to input DNA. ChIP‐qPCR was repeated twice in same condition to confirm the reproducibility of the results.

### 2.7 Immunoprecipitation

Briefly, the lysis buffer (Cell Signaling) was used to lyse the cells, then the lysates were centrifuged to exclude the impurity substance. The protein A/G beads (Sigma) were added into the supernatant and incubated with the specific antibody for 12 h at 4°C. Moreover, the protein A/G beads were incubated with the left immunocomplexes for 2 h at 4°C. The pellets were obtained and washed five times with indicated lysis buffer after centrifugation. Western blot assay was used to detect the potential interacting proteins.

### 2.8 Generation of kidney cancer xenografts in mice

All animal assays are in agreement with ethical requirements and have been approved by the Institutional Animal Care Use Committee of Shanghai RuiJin Hospital, Shanghai JiaoTong University. Besides, 4–6 weeks’ old BALB/c nu/nu mice were obtained from SLAC Laboratory Animal Co., Ltd. After pre‐experiments, 6 × 10^6^ indicated RCC cells were suspended into the 100 μl of PBS buffer and then injected into the armpits of nude mice (five mice per group). All mice were sacrificed and in vivo solid tumors were dissected after 3 weeks.

### 2.9 Establishment of patient‐derived xenograft and patient‐derived organoids models

To obtain the subcutaneous patient‐derived xenograft (PDX) models, fresh human ccRCC tissues were resected from RCC patients. To optimize the tumor take rate of PDX models, we used the original patient specimens containing high amounts of viable cancer, which are defined as samples with (i) notable Ki67 expression, (ii) no remarkable damage, and (iii) ≥50% cancer cells. The minor portion of the tumor was obtained and fixed for BRD9 staining within 6 h of sample arrival. The remaining tumors were resected into smaller pieces (about 1 × 3 × 3 mm^3^) and implanted surgically into the subcutaneous tissue in the flanks of 4‐week‐old BALB/c‐nu mice. The mice were raised in a clean condition at the Shanghai Ruijin Hospital. All initial implants were conducted with 200 μl of Matrigel (BD Bioscience, San Jose, CA) injected around the renal cancer implants. Once a xenograft was passaged two or three times, Matrigel was no longer required for serial propagation. After several subcutaneous passages in mice, the stable ccRCC PDX models were established. To obtain the patient‐derived organoids, the single‐cell suspensions were obtained via enzymatically digesting the fresh ccRCC tissues and then incubated with 200 U/ml of deoxyribonuclease I (Roche, Indianapolis, IN) and collagenase type IV (Sigma, St. Louis, MO) for 1 h at 37°C. The sterile gauze and 100‐mm nylon mesh were used to filter the cells that were plated in Matrigel (BD) and cultured by DMEM/F12 medium containing 1.25 mM *N*‐acetyl‐l‐cysteine, 2% B27, 50  ng/ml EGF, 100 ng/ml Noggin, 200 nM A83‐01, 10 μM Y‐27632, 500 ng/ml R‐spondin 1, and 1 nM dihydrotestosterone. To perform the organoid formation assays, approximately 2000 cells were plated per well on Day 1, and the number and diameter sizes of the organoids were detected and compared on Day 7, Day 14, and Day 21. The specific media composition of organoids is summarized in Table S1.

### 2.10 Bioinformatics and statistical analysis

The other eligible RCC patients were all obtained from the public datasets, including TCGA (https://portal.gdc.cancer.gov/) and ICGC (https://icgc.org/). The differential analysis of mRNA or protein levels were determined via Wilcoxon test. The RNA‐seq data were normalized with edgeR package and matched with complete clinical information. For pan‐cancer analysis, the JMJD6 expression data were extracted from 33 tumors matched with normal samples. The differential analysis of JMJD6 in 33 tumors was determined via Wilcoxon test. LASSO regression, Cox regression analysis, and Kaplan–Meier analysis were mainly conducted by the glmnet and survival packages. Correlation analysis was conducted with corrplot package. The ROSE2 (Rank Order of Super Enhancers) software (https://github.com/BradnerLab/pipeline) was utilized to identify the putative enhancer regions, using distal (>2.5 kb from TSS) H3K27ac peaks. All bioinformatic statistical analysis was mainly performed in R studio (Version 3.6.1). All experiments were carried out in three biological replicates, respectively. Results from three biological replicates were expressed as the mean ± SD. The one‐way ANOVA or two‐tailed Student's *t*‐test was used to analyze the differences between treatment regimens. The *P* < 0.05 was regarded to be of statistical significance. Lastly, *P < 0.05, ** *P* < 0.01, *** *P* < 0.001, and **** *P* < 0.0001.

## 3 RESULTS

### 3.1 GeCK functional screening data and RCC cohorts were integrated to highlight JMJD6 as a fitness candidate for RCC

**FIGURE 1:**
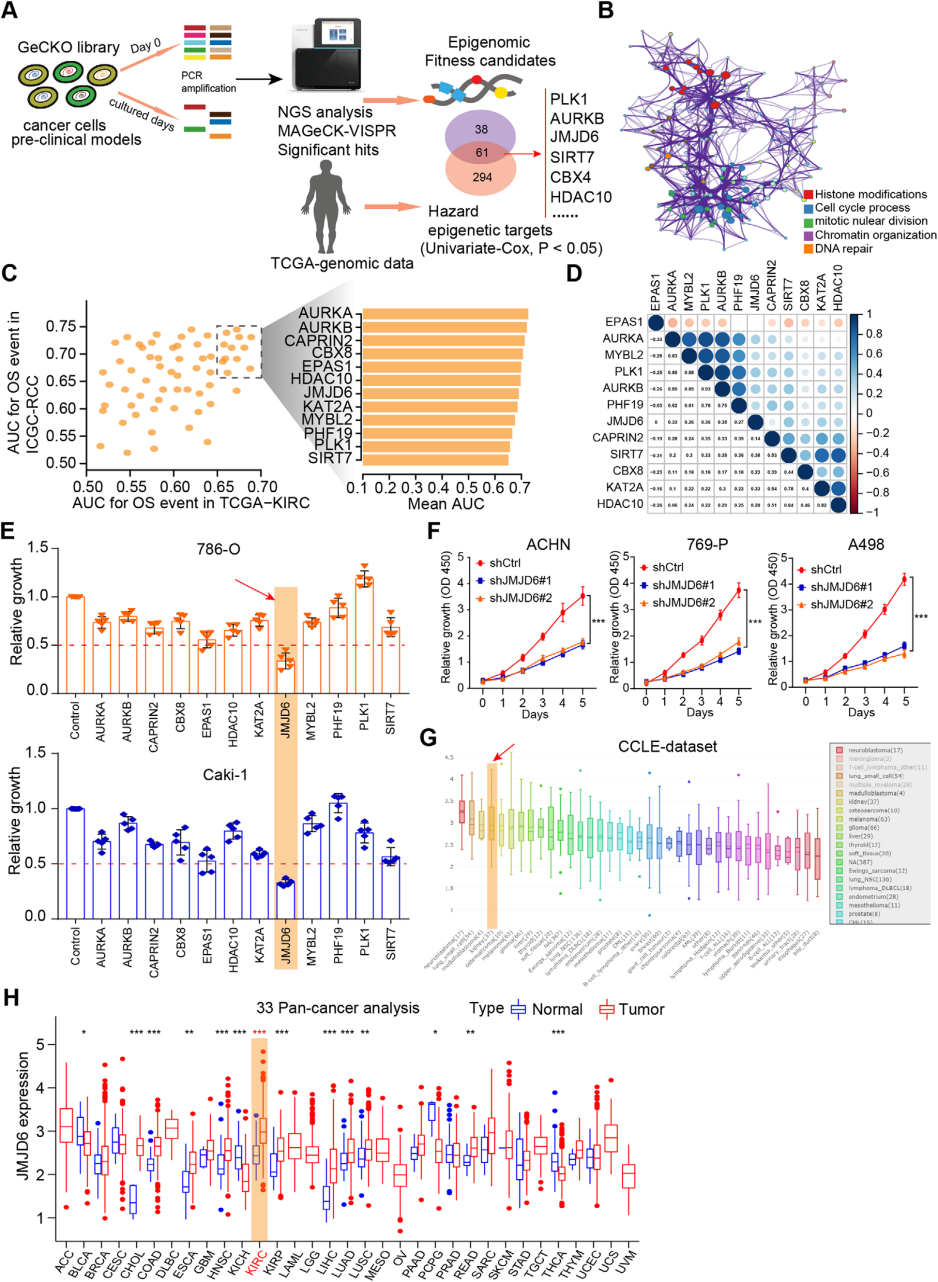
High‐throughput sequencing data and experimental validations identified JMJD6 as a potential epigenetic fitness gene for RCC. (A) Illustrations of the screening strategies incorporating GeCK data and RCC datasets. (B) Functional enrichment revealed several oncogenic cross talk, such as histone modifications, cell cycle processes, chromatin organization, and DNA repair, based on the 61 potential epigenetic fitness genes. (C) The area under the curve (AUC) evaluation of the prognostic value of candidate genes involved in overall survival‐related events based on datasets TCGA‐KIRC and ICGC‐RCC. (D) Besides, we utilized the heatmap to reveal the potential relationships across the 12 prognostic genes. (E) Meanwhile, MTT assay was used to validate the screening results using specific siRNAs and revealed that JMJD6 was the most potent hit in relative to other epigenetic factors in RCC cells. (F) Independent assays further validated that JMJD6 knockdown remarkably suppressed RCC proliferation in three RCC cell lines. (G) The Cancer Cell Line Encyclopedia (CCLE) dataset revealed that JMJD6 was highly expressed in kidney cancer compared with most of other solid tumors. (H) Lastly, the pan‐cancer analysis based on 33 tumors suggested that JMJD6 was highly expressed in RCC patients relative to most of other tumor types

Given that CRISPR–Cas9 screens effectively identify robust targets across cancers, researchers have recently integrated screening data with genomic biomarkers to prioritize new candidates for tumor vulnerability. In particular, we derived a list of 1614 fitness genes in RCC from the comprehensive pan‐cancer results^16^ (Table S2). In addition, we extracted expression data of 665 epigenetic regulators from a TCGA‐KIRC (Kidney Renal Clear Cell Carcinoma) cohort, and univariate Cox regression analysis was used to screen a total of 355 hazardous epigenetic factors at *P* < 0.05. We overlapped the data from the two screening results and ultimately identified 61 potential epigenetic fitness genes in RCC, such as PLK1, AURKB, BRD4, and BPTF (Figure 1A). Gene Ontology (GO) analysis further revealed that the intersecting genes were mainly enriched in histone modifications, cell cycle processes, chromatin organization, and DNA repair (Figure 1B). In addition, we utilized area under the curve (AUC) analysis to evaluate the predictive efficiency of candidate genes based on two independent datasets featuring TCGA‐KIRC and ICGC‐RCC cohort data (Figure 1C; Table S3). The underlying relationships of 12 genes were illustrated in a correlation heatmap (Figure 1D). To further identify the most potent epigenetic regulator associated with RCC growth, we performed an MTT assay to validate the screening results and used individual siRNAs to compare the antitumor effects of specific genes. The MTT assay indicated that JMJD6 was the most promising hit compared with other genes in RCC cell lines (Figure 1E). Furthermore, JMJD6 knockdown remarkably suppressed RCC proliferation in three independent RCC cell lines (Figure 1F). We used the Cancer Cell Line Encyclopedia (CCLE) dataset and observed that JMJD6 was highly expressed in kidney cancer relative to most other solid tumors (Figure 1G). Finally, we conducted the pan‐cancer analysis using the expression data from 33 tumors and found that JMJD6 was highly expressed in RCC patients compared with most of other tumor types (Figure 1H). Taken together, these data suggest that JMJD6 is an essential epigenetic candidate in RCC that deserves to be further investigated.

### 3.2 High JMJD6 level correlated with poor prognosis in RCC patients and could act as an independent marker

**FIGURE 2:**
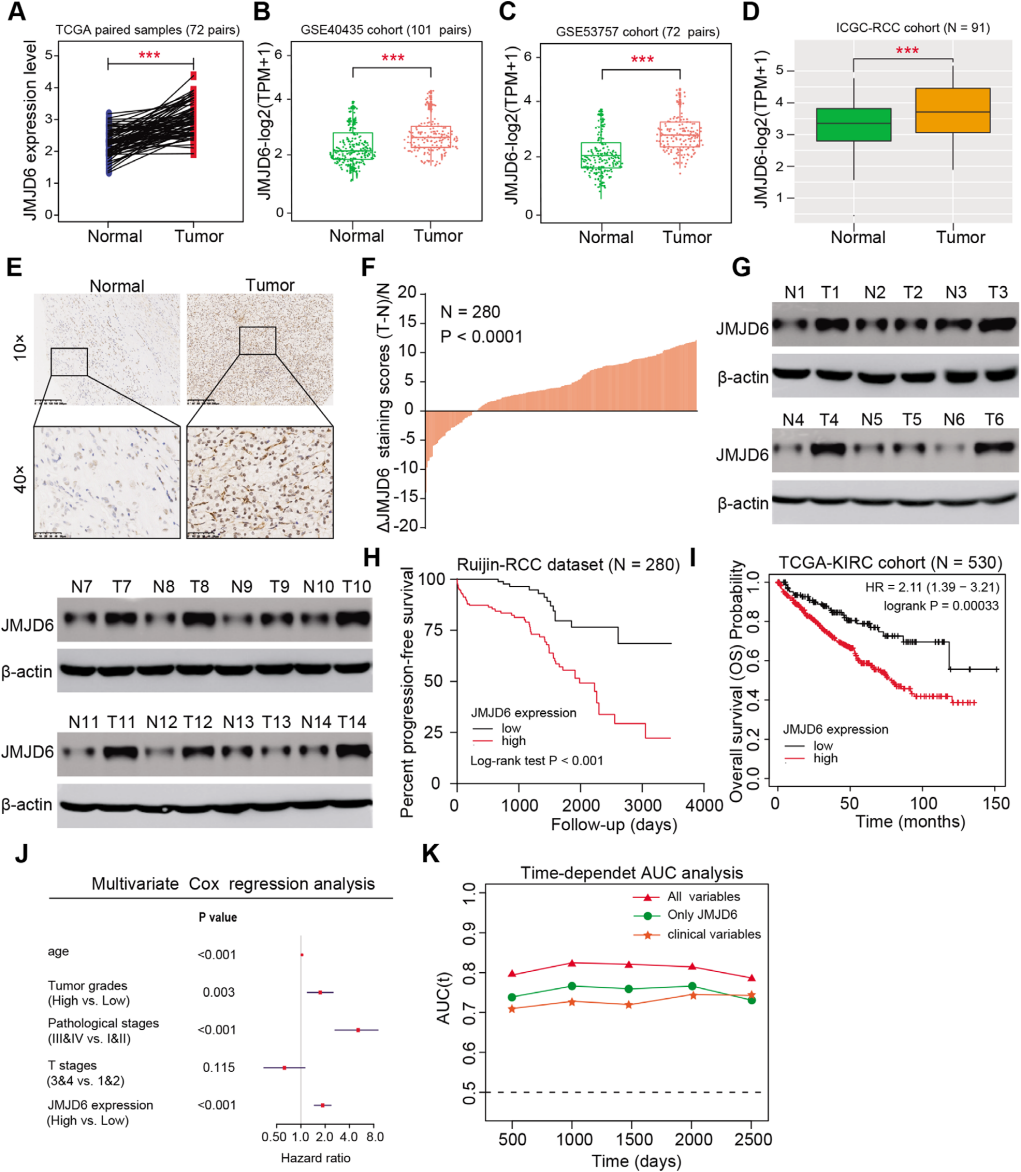
JMJD6 expression level is positively associated with OS of RCC patients and exhibits independent prognostic significance. (A–D) JMJD6 exhibited significantly higher levels in tumor samples versus normal tissues from the multiple RCC datasets, such as TCGA‐KIRC, GSE40435, GSE53757, and ICGC‐RCC cohorts. (E) Representative IHC images of tumor and paired normal sections on the tissue microarray (TMA) probed with the anti‐JMJD6 antibody (scale bars = 200 or 50 μm, respectively) are shown. (F) The distribution of the difference in JMJD6 immunoreactivity score (△scores = (Tumor − Normal)/Normal). The △scores of JMJD6 staining was available in 280 pairs of tissues. (G) Moreover, JMJD6 protein levels were also detected in RCC tissues and paired normal kidney tissues via western blotting assay (*N* = 14). (H) Kaplan–Meier analysis in Ruijin RCC dataset (*N* = 280) suggested that patients with high JMJD6 levels suffered from worse progression‐free survival (PFS) outcomes compared with those with low JMJD6 levels. (I) In line with the previous results, high JMJD6 mRNA levels also correlated with worse overall survival (OS) outcomes based on the analysis of sequencing data of TCGA‐KIRC cohort (*N* = 530). (J) Multivariable analyses were conducted in the RCC cohort, where all bars were corresponded to 95% CIs. (K) The time‐dependent receiver operating characteristic (ROC) analysis for the tumor grades, pathological stages, and the combined JMJD6 levels in the RCC cohort

To further figure out the oncogenic role of JMJD6 in RCC, we analyzed several cohorts to evaluate its clinical significance and found that JMJD6 was commonly higher in tumor samples than in normal tissues from the TCGA‐KIRC cohort, GSE40435, GSE53757, and IGC‐RCC cohort (Figure 2A–D). In addition, we confirmed the results in a microarray assay of RCC samples, in which JMJD6 was significantly elevated in RCC samples compared with paired normal tissue samples, as determined by immunohistochemistry (IHC) (*N* = 280, *P* < 0.001, Figures 2E and 2F). Consistent with these results, the JMJD6 protein levels were remarkably higher in 11 of 14 (78.6%) fresh RCC tissues than in their matched normal tissues, as determined by western blot analysis (Figure 1G). Moreover, based on the analysis of the TCGA‐KIRC cohort, high expression of JMJD6 correlated positively with advanced TM stages, pathological stages, and tumor grades (*P* < 0.01, Figure S1A). Kaplan–Meier analysis further indicated that high JMJD6 levels correlated with worse overall survival (OS) and progression‐free survival for patients from the TCGA‐KIRC cohort and Ruijin‐RCC dataset (Figures 2H and 2I). Simultaneously, we conducted a multivariate Cox analysis using age, pathological stage, and tumor grade and found that JMJD6 still remained one independent predictive marker for the prognosis of RCC (HR = 1.289, 95% CI, 1.136−1.463, *P* < 0.001, Figure 2J). Finally, we conducted a time‐dependent receiver operating characteristic (ROC) curve assessment to evaluate the predictive ability of JMJD6 expression. The combination of clinical risk variables with JMJD6 expression in the TCGA‐KIRC cohort contributed much more than any factor alone (Figure 2K). Taken together, these findings indicated that the JMJD6 level expressed highly in RCC and JMJD6 possessed the potentiality as an independent prognostic factor for RCC patients.

### 3.3 Oncogenic JMJD6 promotes RCC progression in vitro and in vivo

**FIGURE 3:**
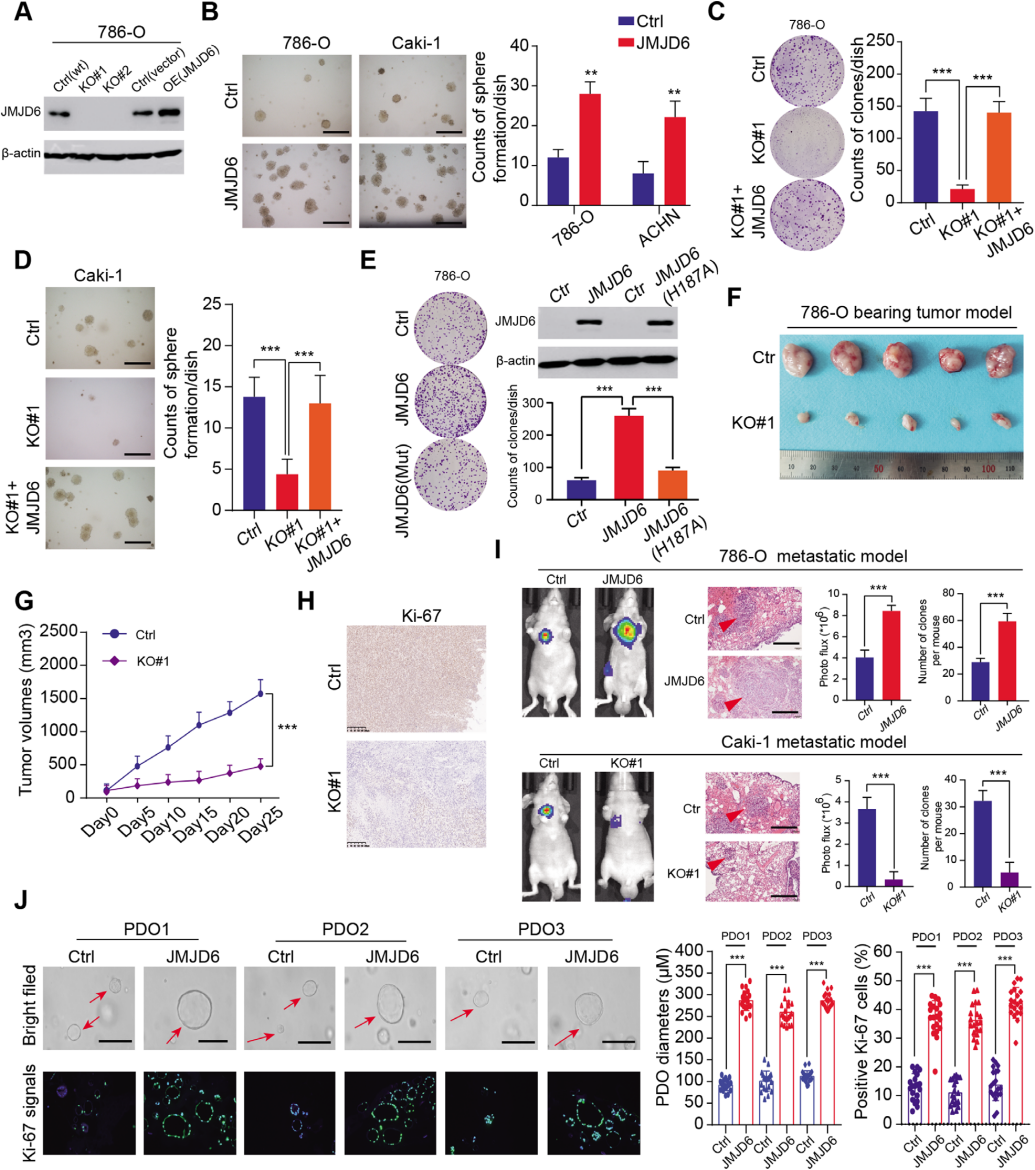
JMJD6 promotes RCC proliferation and distal metastases in vitro and in vivo. (A) To demonstrate the biological roles of JMJD6, we first constructed the stable GFP‐tagged JMJD6‐overexpressing and JMJD6‐knockout RCC cell lines via CRISPR/Cas9 technology, which were validated by western blotting. (B) JMJD6 overexpression promoted RCC cell (786‐O and Caki‐1) anchorage‐independent growth in soft agar (scale bars = 200 μm). Quantification of the soft agar colony formation assay results (right panel). (C–D) Besides, JMJD6 knockout could notably suppress the RCC colony formation ability, which could be rescued via restoration of JMJD6 levels. (E) However, ectopic expression of inactive JMJD6 mutant (H187A) failed to rescue the suppressive effect induced by JMJD6 deficiency. (F) JMJD6 deficiency significantly inhibited RCC subcutaneous tumor growth in nude mice (*N* = 5). (G) The tumor volume was detected at indicated days, and tumor growth curves were generated. (H) The sections of tumors from two groups were collected and stained with anti‐Ki‐67 via IHC, where the scale bars = 200μm. (I) Furthermore, overexpressed JMJD6 enhanced RCC distal metastases, whereas JMJD6 knockout significantly impaired the tumor metastatic ability, as indicated by bioluminescence signals and lung metastatic node numbers. (J) The representative pictures of three independent RCC organoids transfected with JMJD6 overexpression vectors or control lentivirus for 2 weeks were shown (scale bars = 250 μm, left panel) and quantified via organoid diameters (right panel)

To further examine the biological function of JMJD6 in RCC, stable GFP‐tagged JMJD6‐overexpressing or JMJD6‐knockout RCC cell lines (786‐O and Caki‐1) were established (Figures 3A and S2A). The RCC cell colony formation efficiency in soft agar was significantly promoted in the JMJD6‐overexpressed group compared with that in the control group (Figure 3B). In addition, JMJD6 deficiency significantly impeded RCC growth (786‐O and Caki‐1), which was completely rescued through the restoration of exogenously expressed JMJD6 tagged with FLAG (Figures 3C and 3D). However, the well‐known JMJD6(H187A) mutant causing enzymatic deficiency remarkably impaired the formation of RCC clones compared to those formed by the JMJD6(WT) group^37^ (Figure 3E). Flow cytometry analysis further revealed that JMJD6 deficiency exerted a cytostatic effect and promoted cell apoptosis, in accordance with its oncogenic features in RCC (Figure S2C‐D). In addition, to determine the roles of JMJD6 in vivo, we performed tumor xenograft studies and observed that knocking out JMJD6 significantly suppressed tumor growth, as quantified by tumor size, compared with tumors derived from control group (Figures 3F and 3G). In addition, the IHC results further revealed that Ki‐67, an indicator of proliferation, was notably decreased in tumors derived from JMJD6‐KO cells (Figure 3H). Furthermore, we also detected that ectopic expression of JMJD6 enhanced the migration and invasion of 786‐O and Caki‐1 cells (Figure S2B). We assessed the effect of JMJD6 on RCC metastasis in vivo and injected luciferase‐tagged RCC cells into the splenic portal vein of nude mice. As quantified by bioluminescence signals and number of lung metastatic nodes, JMJD6 overexpression remarkably promoted RCC lung metastasis, whereas JMJD6 deficiency dramatically inhibited the metastatic ability of RCC tumors to metastasize to the lung relative to the corresponding control groups (Figure 3I). Finally, an RCC‐specific organoid model was established from three different patients, and we observed that the lentivirus‐mediated overexpression of JMJD6 markedly promoted RCC cell proliferation in three independent RCC organoids, as quantified by organoid sizes and Ki‐67 signal intensity (Figure 3J). Collectively, these data suggested that JMJD6 can function as a robust oncogene that promotes RCC proliferation.

### 3.4 p300‐mediated H3K27ac activates JMJD6 transcription in RCC

**FIGURE 4:**
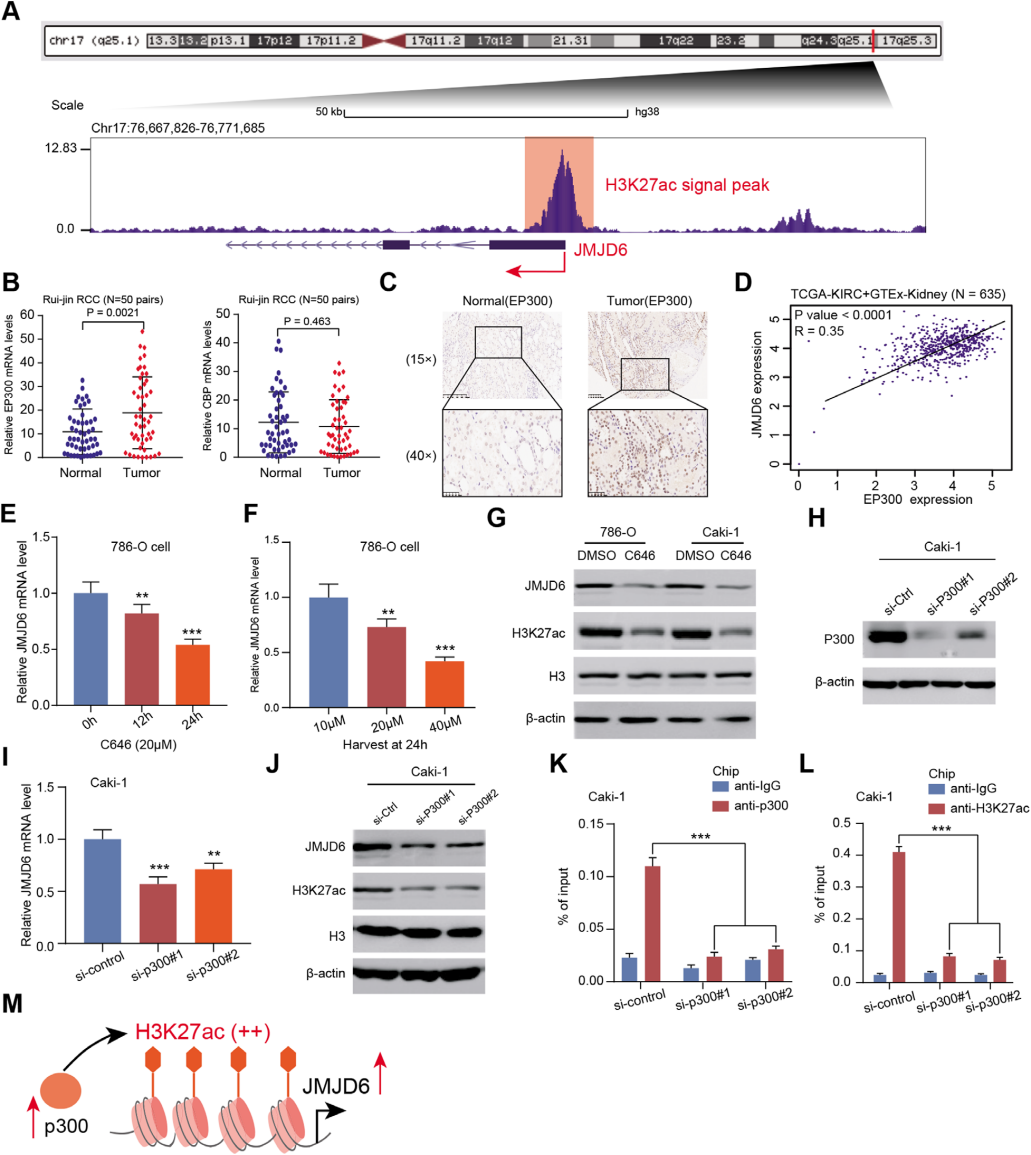
The p300‐mediated H3K27ac activates JMJD6 transcription in RCC. (A) We screened and observed the high enrichment of H3K27ac at the promoter region of JMJD6 from the data of the UCSC genome bioinformatics site (http://genome.ucsc.edu/). (B) We compared and found that p300 mRNA levels exhibited significantly higher in tumors than that in normal tissues, whereas no notable CBP alterations were found. (C) The IHC assay further validated that p300 was higher in tumor tissues versus normal sections, where the scale bars were 200 and 50 μm, respectively. (D) Besides, a remarkably positive correlation between p300 and JMJD6 mRNA levels was calculated with *P* < 0.0001 based on the analysis of TCGA‐KIRC and GTEx data. (E and F) C646, a histone acetyltransferase inhibitor targeting p300, was selected to treat RCC cells, and a significant decrease of JMJD6 mRNA levels was observed in a time‐ and dose‐dependent manner. (G) The JMJD6 and H3K27ac protein levels in RCC cell lines (786‐O and Caki‐1) were detected via western blotting with C646 treatment (20 μM) for 24 h. (H) The specific siRNAs targeting p300 were designed and the knockdown efficiency was confirmed via western blotting. (I) The quantitative real‐time polymerase chain reaction (qRT‐PCR) method was used to detect the JMJD6 expression levels in Caki‐1 cells when knocking down the p300. (J) Both the protein levels of JMJD6 and H3K27ac were decreased significantly after the p300 knockdown. The levels of p300 binding (K) and the enrichment of H3K27ac at the promoter region of JMJD6 (L) in p300 knockdown or control Caki‐1 cells were determined via ChIP assays. (M) The simple graphical illustration of the mechanisms was drawn to show that p300‐mediated H3K27ac activation enhances JMJD6 transcription

To investigate the underlying mechanisms that mediate high JMJD6 expression in RCC, we first considered the transcriptional regulation or modification of the promoter of JMJD6 using the Cistrome Data Browser, a comprehensive epigenetic dataset (http://cistrome.org/db/#/). We detected and observed that H3K27 acetylation (H3K27ac) peaks, well‐defined markers of active enhancers and transcription, were significantly abundant at the promoter of JMJD6, indicating a potential role for chromatin acetylation in JMJD6 regulation (Figure 4A). As previously reported, the p300/CPB complex mediates the catalytic process of cellular acetylation of H3K27ac, which directs H3K27me loss and reciprocal H3K27ac gain.^37^, ^38^ Accordingly, we found that p300 protein levels were significantly higher in RCC than in normal tissues (*P* = 0.0021), whereas CBP expression was not different between RCC and normal kidney tissues (*P* = 0.463) (Figure 4C). These findings were further validated in a microarray assay using IHC (Figure 4D). By analyzing the TCGA‐KIRC and GTEx data, we found a remarkably positive correlation between EP300 and JMJD6 mRNA levels with *P* < 0.0001 (Figure 4D). C646, a histone acetyltransferase inhibitor targeting p300, was selected to treat RCC cells, and we found a significant decrease in JMJD6 mRNA levels in a time‐ and dose‐dependent manner (Figures 4E, 4F, and S2E). Performing western blot analysis, we found that the protein levels of JMJD6 and H3K27ac were reduced synchronously in 786‐O and Caki‐1 cells treated with C646 (Figure 4G). We thus designed two specific siRNAs targeting p300 (Figure 4H). As expected, knocking down p300 resulted in considerable reductions in both the mRNA and protein levels of JMJD6, along with the H3K27ac levels (Figures 4I and 4J). Furthermore, we selected another chromatin modifier CHD6 as the negative control and repeat the above assays again. Intriguingly, we did not observe significant abundance of H3K27ac peaks at the CHD6 promoter region (Figure S2F). Neither C646 nor p300 knockdown could reduce the expression levels of CHD6 in RCC lines, suggesting the specific effect of p300 on CHD6 (Figure S2G‐J). Finally, ChIP analysis was performed to verify that H3K27ac signals and p300 binding were both enriched at the promoter regions of JMJD6, and p300 ablation significantly weakened the enrichment of H3K27ac signals, in line with previous findings (Figures 4K and 4L). Taken together, these results indicate that the aberrant expression of p300 and subsequent p300‐mediated H3K27ac modification may partially result in and explain the high JMJD6 expression levels in RCC (Figure 4M).

### 3.5 Overexpressed JMJD6 alters the oncogenic transcriptome in RCC cells to promote tumorigenesis

**FIGURE 5:**
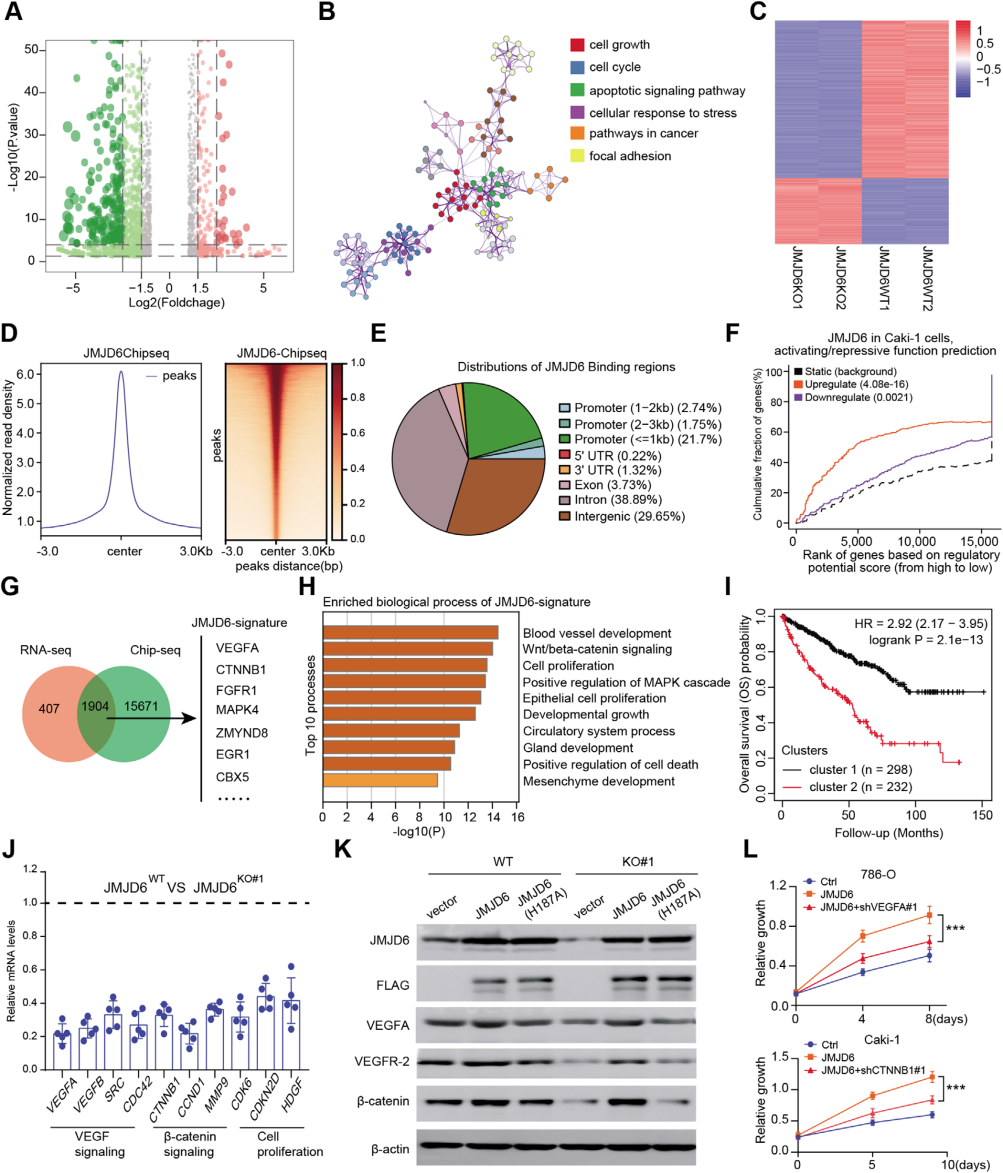
Overexpressed JMJD6 alters the oncogenic transcriptome in RCC cells to promote tumorigenesis. (A) The differential analysis was conducted in JMJD6^WT^ and JMJD6^−/−^ cells and the differentially expressed genes (DEGs) were exhibited in volcano plot. (B) The Gene Ontology (GO) analysis indicated that the top 300 DEGs were significantly enriched in cell growth, cell cycle, apoptotic signaling pathways, and pathways in cancer categories. (C) The cluster of DEGs was shown in heatmap. (D) ChIP‐Seq summary plot of JMJD6‐binding intensities across JMJD6 peaks in Caki‐1 cells. (E) The distributions of JMJD6‐binding regions were shown in the pie charts. (F) The binding and expression target analysis (BETA) suggested that JMJD6 plays an active factor in Caki‐1 cells, where the red and dark purple lines indicate upregulated and downregulated genes, respectively. (G) We overlapped the RNA‐seq data and Chip‐seq data to narrow down the JMJD6 signature in RCC, indicated by Venn diagram. (H) The Gene Ontology (GO) analysis revealed the significantly enriched items based on JMJD6 signature. (I) The JMJD6 signature can successfully stratify the data of patients via K‐means clustering into two clusters for overall survival (*P*‐values by log‐rank test). (J) quantitative real‐time polymerase chain reaction (qRT‐PCR) analysis of gene expressions in JMJD6 intact and knockout cells. (K) The western blotting assay was used to further demonstrate the associations among JMJD6 and downstream targets. (L) VEGFA or β‐catenin knockdown via specific shRNAs markedly attenuated JMJD6‐induced RCC growth in vitro

To determine the underlying mechanism by which JMJD6 promotes RCC growth and progression, we conducted transcriptome profiling analysis of JMJD6‐WT and JMJD6‐knockout cells. The analysis revealed 2311 differentially expressed genes (DEGs) between the two groups, of which 682 genes were upregulated and 1629 genes were downregulated upon JMJD6 ablation (Figure 5A; Table S4). A GO analysis suggested that the top 300 DEGs were significantly enriched in cell growth, cell cycle, apoptotic signaling pathways, and pathways in cancer categories, implying an oncogenic role of JMJD6 in RCC (Figure 5B). We also generated a heatmap to observe the DEGs in clusters (Figure 5C). To identify definitive downstream targets regulated by JMJD6, we performed ChIP‐seq and found a total of 56,531 peaks comparedwith input signals. The JMJD6‐binding distributions are shown in the pie charts (Figure 5D‐E; Table S5). In line with previous studies, JMJD6 mainly binds to distal enhancer regions. Subsequent binding and expression target analysis (BETA) suggested that JMJD6 mainly functioned as an activation factor in Caki‐1 cells, and the red and dark purple lines indicated upregulated and downregulated genes, respectively (Figure 5F). To screen the putative targets of JMJD6 in RCC, we overlapped the two omics datasets (RNA‐seq, Chip‐seq) to identify 1904 intersecting genes, which were defined as the JMJD6 signature, including well‐known kidney cancer drivers such as vascular endothelial growth factor A (VEGFA), β‐catenin, FGFR1, and MAPK4 (Figure 5G; Table S6). Accordingly, we illustrated several JMJD6‐binding peaks in representative target gene loci (Figure S2K). Subsequently, the GO analysis indicated that blood vessel development was the most enriched cross‐talk function, followed by Wnt/beta‐catenin signaling, cell proliferation, and positive regulation of the MAPK cascade (Figure 5H). Furthermore, we found that the JMJD6 signature can successfully stratify the data of patients via K‐means clustering into two clusters that exhibit distinct differences in terms of OS (log‐rank test, *P* < 0.001, Figure 5I). Although it was difficult to attribute the JMJD6 oncogenic effect to a segmented target gene, we selected several top genes known to be essential for RCC progression and measured their expression levels. As a result, genes representative of pivotal drivers of VEGFA signaling, β‐catenin signaling, and cell proliferation in RCC were chosen, including VEGFA/B, SRC proto‐oncogene, nonreceptor tyrosine kinase (SRC), CDC42, CTNNB1, CCND1, MMP9, CDK6, CDKN2D, and HDGF (Figure 5J). The western blot assay further demonstrated that wild‐type JMJD6, but not the inactive JMJD6 mutant (H187A), maintained the high levels of VEGFA crosstalk and β‐catenin and could reverse the impaired effects induced by JMJD6 deficiency (Figure 5K). Moreover, VEGFA or β‐catenin knockdown markedly impaired JMJD6‐induced RCC growth in vitro (Figure 5L). Collectively, our data indicated that JMJD6 initiates a tumorigenic transcriptome profile involving several oncogenic cross‐talking pathways, particularly the VEGFA signaling.

### 3.6 JMJD6 constitutes super‐enhancers to drive downstream targets such as VEGFA and β‐catenin

**FIGURE 6:**
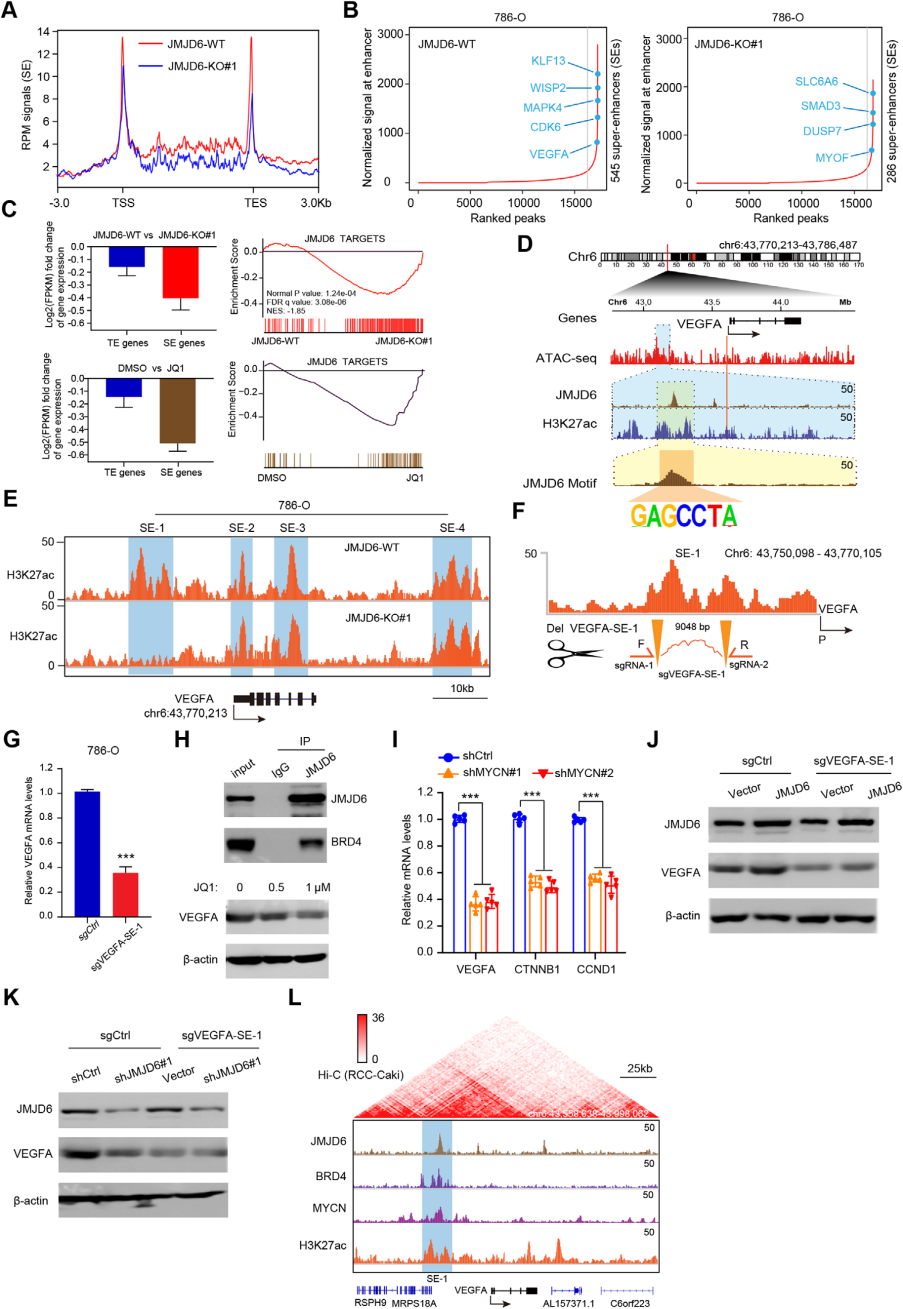
JMJD6 mainly constitutes super‐enhancers to drive RCC identity targets such as VEGFA. (A) The differential enhancer landscapes between JMJD6‐deficient and control cells were compared based on the ChIP‐seq data of H3K27ac. (B) The ROSE algorithm was utilized to detect and compare the differential SEs in JMJD6 intact and JMJD6‐knockout cells. (C) Selective disruption of SE genes upon JMJD6 knockout or BET bromodomain inhibition (JQ1) in Caki‐1 cells (Left). Error bars show the 95% confidence interval. *P*‐values calculated by Welch's unpaired *t*‐test. Gene set enrichment analysis (GSEA) revealed the inhibition of JMJD6 targets, along with by JQ1 (Right). (D) The multiple tracks in the IGV diagram exhibited the co‐occupancy of JMJD6 and H3K27ac at the VEGFA enhancer region. (E) Comparing the differential H3K27ac peaks near VEGFA in JMJD6‐intact and JMJD6‐deficient cells, we identified one putative SE mediated by JMJD6. (F) Schematic presentation of location of VEGFA as well as the design of CRISPR/Cas9‐mediated deletion of the super‐enhancer VEGFA‐SE‐1. The location of sgRNAs and the primers used to validate the deletion were indicated. (G) Real‐time PCR was used to determine the mRNA level of VEGFA in sgVEGFA‐SE‐1 cells and control cells. Data are represented as mean ± SD. (H) The interaction of JMJD6 and BRD4 was verified by immunoprecipitation (IP) assay and the reduced VEGFA expression levels were determined by western blotting after BRD4 inhibitors (JQ1) treatment. (I) The mRNA expression levels of representative JMJD6 signature were detected and compared via qPCR in MYCN knockdown and control cells. (J) Overexpressed JMJD6 could not rescue the VEGFA expression levels in sgVEGFA‐SE‐1 cells. (K) No altered VEGFA expression levels were detected in sgVEGFA‐SE‐1 cells upon JMJD6 knockdown versus control. (L) The combination of Hi‐C data from the ENCODE dataset with the JMJD6 and coactivator tracks revealed the potential physical interactions between the SE‐bound proteins (N‐MYC, JMJD6, and BRD4) with the promoter of VEGFA to facilitate transcription

Previous studies have reported that JMJD6 can alter enhancer profiles and interact with BRD4 and N‐MYC, which are mainly super‐enhancers (SEs) driving downstream oncogenic signatures. To determine and demonstrate the potential effect and regulation of JMJD6 on targets, we conducted ChIP‐seq of H3K27ac, an enhancer indicator, and compared the differential enhancer landscapes between JMJD6‐deficient and control cells. We observed a minor reduction in H3K27ac signals across the genome upon JMJD6 knockout (Figure 6A). Intriguingly, we utilized the rank oriented of super enhances (ROSE) algorithm to detect SE peaks and found a remarkable reduction in SE signals: there were 545 SEs in control cells and only 286 SEs in JMJD6^−/−^ cells (Figure 6B). Furthermore, we conducted RNA‐seq to compare the fold change of SEs compared to SE‐associated genes upon JQ1 treatment for 8 h. We observed that SE‐associated genes in 786‐O cells were particularly sensitive to JQ1, an observation also noted upon JMJD6 depletion (Figure 6C). Gene set enrichment analysis (GSEA) further indicated that JQ1 can selectively downregulate JMJD6 signature genes with enrichment mirroring the knockout of JMJD6 (Figure 6C). Taking VEGFA as an example, we conducted ATAC‐seq and integrated the multiple tracks in the IGV diagram, where JMJD6 and H3K27ac were found to co‐occupy the VEGFA enhancer region (Figure 6D). We also compared the different H3K27ac peaks near VEGFA in JMJD6‐intact and JMJD6‐deficient cells to identify putative SEs mediated by JMJD6. Intriguingly, there was a remarkable reduction in the H3K27ac signal at one of the SE regions where JMJD6 was also bound (Figure 6E). To test this putative JMJD6‐comprised SE, we utilized the CRISPRi method to target putative JMJD6‐binding sites within this region, and the results indicated an ∼60% reduction in VEGFA expression (Figures 6F and 6G). Considering that JMJD6 interacts with BRD4 to form SEs, we treated RCC cells with a BRD4 inhibitor and found that JQ1 largely reduced VEGFA levels in a dose‐dependent manner (Figure 6H). Besides, targeting MYCN, another JMJD6 co‐activator, could also result in decreased levels of representative JMJD6 targets compared with control group, recapitulating the similar effect of JMJD6 or BRD4 inhibition (Figure 6I). In addition, JMJD6 had no effect on the restoration of VEGFA levels induced by sgVEGFA‐SE‐1 (Figure 6J). Similarly, there were no detectable alterations in VEGFA levels in sgVEGFA‐SE‐1 cells upon JMJD6 knockdown (Figure 6K). Collectively, VEGFA‐SE1 depletion can cause VEGFA to escape from JMJD6 manipulation. Finally, we downloaded the Hi‐C data of RCC cells from the ENCODE dataset (https://www.encodeproject.org/) and integrated the JMJD6 and coactivator tracks, where these loops enable the physical interaction of the SE‐bound proteins N‐MYC and BRD4 with the promoter of VEGFA to facilitate transcription (Figure 6L). Using this strategy, we also identified several putative SE regions in other JMJD6 signatures, such as β‐catenin and SRC (Figures S3A and S3B). Taken together, these data concluded that JMJD6 mainly interacts with BRD4 to constitute SEs that alter downstream targets in RCC, such as VEGFA.

### 3.7 JMJD6 inhibitor (SKLB325) exhibits remarkable antitumor efficacy and mediates sunitinib sensitivity in RCC

**FIGURE 7:**
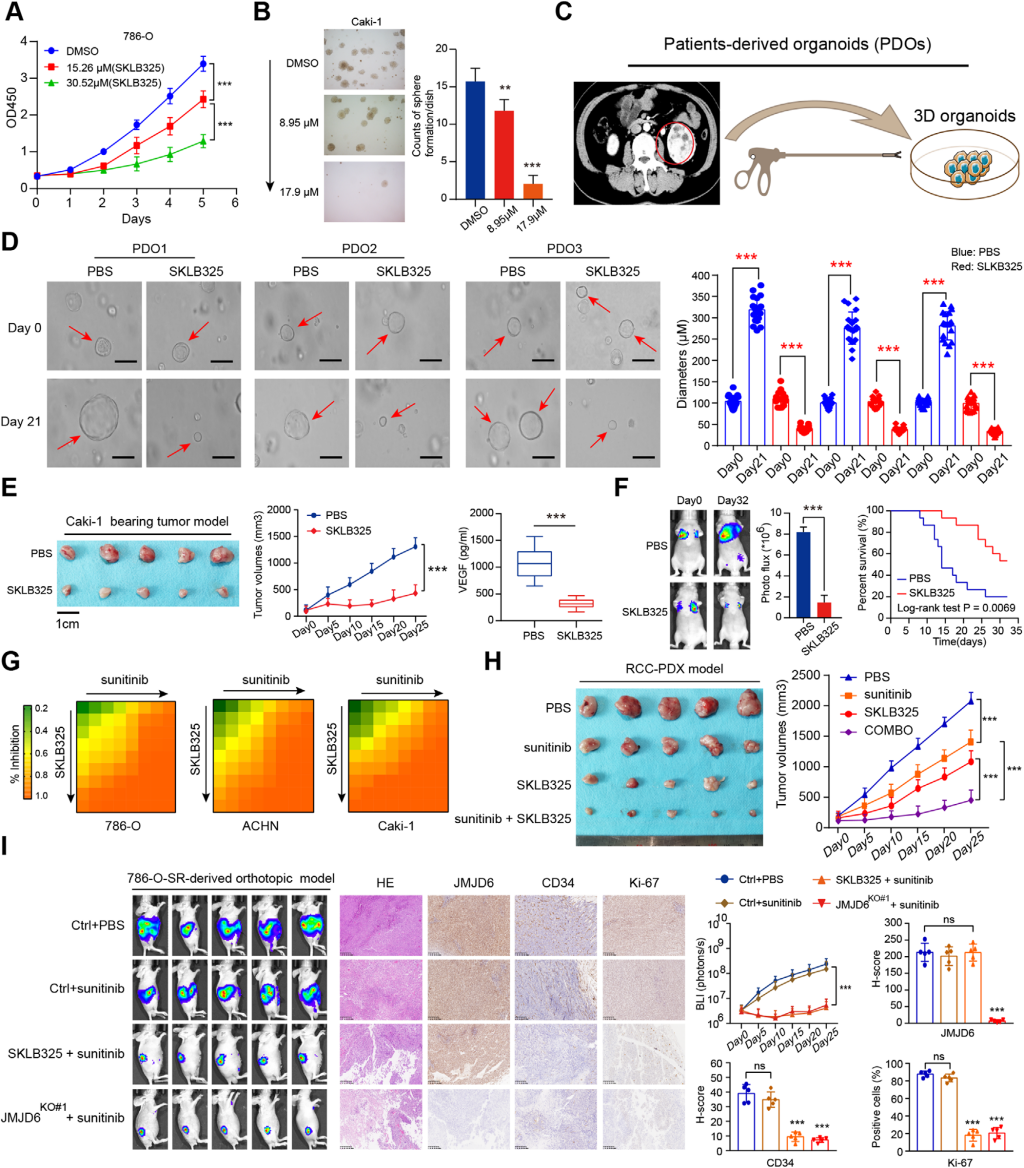
JMJD6 inhibitor (SKLB325) exerts remarkable antitumor efficacy and influences sunitinib sensitivity in RCC. (A) The CCK‐8 assay revealed the attenuated proliferation ability of 786‐O cells caused by SKLB325 in different doses. (B) The soft agar colony formation assay also indicated that the SKLB325 could inhibit the Caki‐1 clonogenic ability in a dose‐dependent manner. (C) The graphical illustration of patient‐derived organoids models derived from RCC patients via laparoscopic resections. (D) Representative images of three different RCC organoids treated with SKLB325 and control PBS for 3 weeks. The diameters of organoids in two groups were compared and calculated as mean ± SD (scale bar = 250μm). (E) SKLB325 effectively inhibited RCC subcutaneous tumor growth in nude mice (*n* = 5), where the tumor volume was monitored at indicated days, and tumor growth curves were generated and compared (middle panel). The serum VEGF concentrations in treated mice and control mice were detected (right panel). (F) Besides, SKLB325 could significantly suppress the distal metastatic ability of RCC cells via tail vein injection, as indicated by Photo flux signals (middle panel), and improved the overall survival of mice (*n* = 15, log‐rank test *P* < 0.01). (G) The inhibitory concentration values for drug combinations (SKLB325 and sunitinib) were shown in heatmap, illustrating the synergistic efficacy. (H) Representative tumors at necropsy (left) and changes in tumor volume (right) of the subcutaneous PDX models treated with PBS control, sunitinib, SKLB325, and combination of both once daily for 28 days (*n* = 10 mice per group). (I) Representative images of the luciferase intensity and orthotopic xenografts from different groups are presented (*n* = 5/group, left panel). Representative images of H&E and IHC staining for JMJD6, CD34, and Ki‐67 in tumor specimens from mice in the four groups were exhibited (scale bar = 200 μm, middle panel). The serial photon flux levels and H‐scores of indicated genes in four groups were calculated and compared (right panel)

Recently, targeting cancer‐associated SEs, such as JQ1, BETis, or THZ1, has become a priority strategy to suppress tumors. However, these drugs work across a broad spectrum of targets and induce multiple side effects. Given the essential roles of JMJD6 in regulating VEGA and other RCC identity drivers, we sought to determine the clinical utility of JMJD6 inhibitors in RCC. First, we synthesized the SKLB325 as instructions and the specific chemical structure was determined via nuclear magnetic resonance spectroscopy in Figure S4A. The half maximal inhibitory concentration (IC_50_) values of SKLB325 were detected in three RCC cell lines (Figure S4B). Then, we observed that the proliferation efficiency and clone formation ability of the RCC cells (786‐O; Caki‐1) were both significantly impeded upon SKLB325 treatment in a dose‐dependent manner (Figures 7A and 7B). To closely imitate the tumor microenvironment and assess the clinical utility of drugs, we established an RCC organoid model with organoids from three RCC patients to further determine the antitumor effect of SKLB325 (Figure 7C). As determined by the sizes of the organoids, SKLB325 was observed to significantly suppress RCC organoid growth relative to the control PBS treatment (Figure 7D). In addition, we also found that SKLB325 remarkably suppressed in vivo tumor size and reduced tumor‐derived VEGF level in the circulation (Figure 3E). Animals were treated via intraperitoneal injection (i.p.) with either SKLB325 (12.5 mg/kg) or PBS (*n* = 8 per group) three times per week for 5 weeks. No adverse reactions were found. In the 786‐O cell‐bearing metastatic model, we confirmed that SKLB325 can also inhibit the growth of distal lung metastases, as quantified by serial bioluminescence signals and metastatic nodes in the lung (Figures 7G and S4C). Kaplan–Meier analysis suggested that mice in the SKLB325 group benefited from a more favorable prognosis than those in the PBS control group (*n* = 15, log‐rank test, *P* = 0.0069, Figure 7F). Sunitinib is a clinically first‐line drug used for advanced RCC that targets multiple RTKs. Nevertheless, a fraction of patients exhibit resistance after a period of sunitinib treatment. Mechanisms of resistance to sunitinib therapy fall into several categories, and the main cause is the bypassed activation of VEGF signaling via other pathways. Given the robustness of the JMJD6/VEGFA axis, we reasoned that JMJD6 status modulated sunitinib efficacy. In line with expectations, we found that overexpressed JMJD6 significantly attenuated sunitinib efficacy compared with that observed in the control group, whereas JMJD6 depletion sensitized RCC cells to sunitinib (Figure S4D). Additionally, we calculated the inhibitory concentration values for drug combinations (SKLB325 and sunitinib) and illustrated the synergistic effect using a heatmap (Figure 7G). To assess the in vivo inhibitory efficacy of SKLB325 in RCC, we further established patient‐derived tumor xenograft (PDX) models from Ruijin‐RCC patient specimens after several subcutaneous passages. In agreement with our previous results using xenograft‐bearing mice, SKLB325 therapy resulted in remarkable tumor regression that was synergistic with sunitinib treatment, as indicated by serial tumor volumes (Figure 7H). Finally, we constructed orthotopic xenograft models using luciferase‐tagged sunitinib‐resistant (SR) cells. No significant difference in tumor growth was found between the sunitinib‐treated SR mice and PBS‐treated mice, indicating the resistance features of 786‐O cell‐SR‐derived orthotopic ccRCC in vivo. Intriguingly, SKLB325 treatment and JMJD6 deficiency exerted a remarkable reduction in tumor growth relative to that of the sunitinib‐treated 786‐O cell‐SR or naive 786‐O cell‐SR mice, as quantified by serial bioluminescence signals (Figure 7H). Additionally, IHC validated that the orthotopic xenografts derived from JMJD6‐deficient or SKLB325‐treated mice exhibited significantly decreased CD34, CD105, and Ki‐67 expression levels (angiogenesis and proliferation markers) compared with that of the other two groups (Figures 7I and S4E). Taken together, these data indicated that a JMJD6 inhibitor (SKLB325) exhibits a significant antitumor effect on primary tumors and distal metastases. High JMJD6 levels might predict natural resistance to VEGF signaling inhibitors, and the combination of SKLB325 with sunitinib was shown to synergistically suppress RCC growth.

**FIGURE 8:**
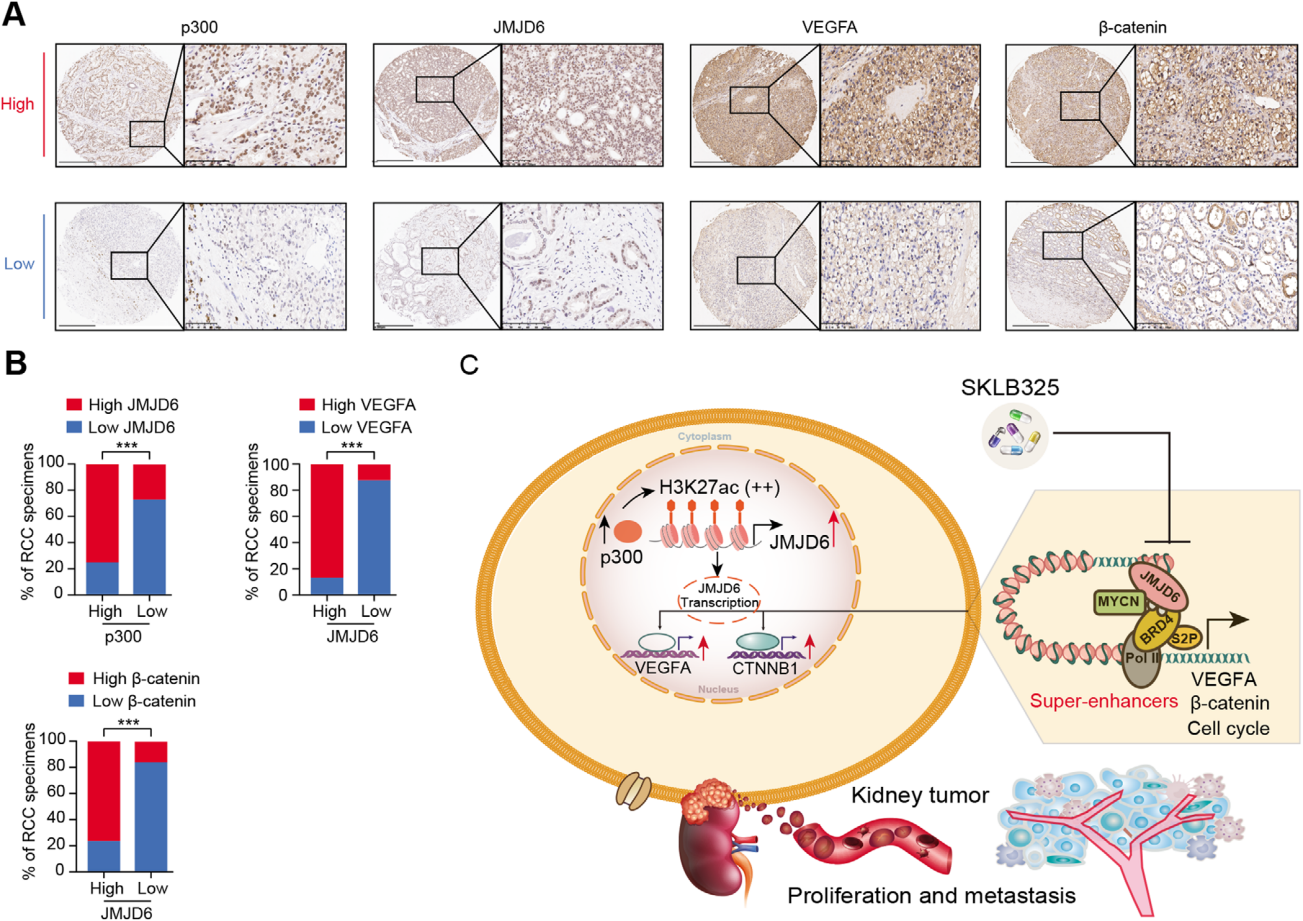
p300–JMJD6–VEGFA/β‐catenin axis is activated in RCC. (A) Immunohistochemical staining of p300, JMJD6, VEGFA, and β‐catenin expression in RCC patients (scale bars = 300 and 100 μm, respectively). (B) The percentages of RCC specimens showing low or high JMJD6 expression relative to the levels of p300, VEGFA, and β‐catenin are shown. (C) The graphic illustration of JMJD6 constituting super‐enhancers to drive downstream targets that promote tumor proliferation and distal metastasis of RCC

To confirm the above p300/JMJD6 axis and JMJD6 downstream targets in clinical samples, we detected the expression levels of p300, JMJD6, and representative JMJD6‐driving targets (VEGFA and β‐catenin) using the RCC tissue microarrays from Ruijin Hospital. Subsequently, we categorized the RCC tissues into the p300‐low and p300‐high groups according to the median H‐score data of 34.8 and determined their expression relevance in RCC (Figure 8A). The expression of JMJD6 was positively associated with the expression of p300, VEGFA, and β‐catenin in the 70 RCC patients (Figure 8B). Furthermore, we comprehensively illustrate the aberrant p300/JMJD6 axis in promoting RCC progression, at least partly through inducing VEGFA, β‐catein, or other JMJD6 signature (Figure 8C).

## 4 DISCUSSION

Aberrant expression levels or truncation mutations in chromatin modifiers have been recognized as vital pathological features in RCC.^3^, ^39^ In addition to the loss of function of canonical divers (*VHL*, *SETD2*, or *BAP1*), essential epigenetic vulnerabilities may contribute to RCC proliferation and may be developed into drug targets. In the current study, we combined the GeCK screening data and TCGA cohort data to identify a cluster of epigenetic fitness candidates, in which JMJD6 was the pivotal hit via functional validations. JMJD6, activated by p300, is highly expressed in RCC and was confirmed as an independent prognostic factor across multiple RCC datasets. In addition, JMJD6 promoted RCC growth and distant lung metastasis in vitro and in vivo. Mechanistically, JMJD6 altered a series of oncogenic signatures and mainly activated blood vessel development and β‐catenin signaling crosstalk. In particular, JMJD6 was demonstrated to be a robust SE that drives VEGFA expression levels. Finally, we assessed the efficacy of a novel JMJD6 inhibitor (SKLB325) for suppressing RCC progression and observed its synergistic efficacy when combined with the first‐line drug sunitinib.

Recently, researchers have conducted CRISPR/Cas9 screening of a panel of cancer cell lines and considered genomic and tractability data to comprehensively identify novel drug targets through an unbiased tool. Unbiased strategies may expand the scope of druggable targets in cancer and accelerate the development of new treatment therapies.^20^ The GeCK library, epigenomic library, and kinase library have been successfully used to screen and identify potential targets in various cancers associated with cisplatin resistance, immunotherapeutic sensitivity, or metastasis.^40^, ^41^, ^42^ The functional library screens can largely prevent missing targets caused by false‐negative results obtained from previous genetic transcription screens, such as microarrays, as some essential genes may exhibit no altered expressions in different conditions. Therefore, we extracted and focused on epigenetic candidates in RCC cells based on the GeCK results. Furthermore, in line with the emerging hotspot of PCAWG, high‐throughput screening of large cohorts (TCGA/ICGC) has become a powerful method to identify the underlying prognostic factors in cancer. Complementary to each other, these two screening strategies were combined together to identify JMJD6 as the pivotal epigenetic vulnerability in RCC, which had never been investigated in RCC. Nevertheless, although the utilization of these two screening methods enabled the range of candidates to be narrowed to a large extent, low‐throughput validations via experimental assays are warranted for follow‐up, owing to the proportions of potentially false‐positive targets.

As reported, JMJD6 possesses double functions as a histone arginine demethylase and hydroxylase, dynamically regulating chromatin and gene transcription. Particularly, JMJD6 could interact with BDR4 physically and functionally to modulate Pol II promoter proximal pause release of a large cohort of genes, especially remodeling the enhancer profiles.^29^, ^36^ Previous studies have indicated that JMJD6 overexpression may result in poor prognosis for multiple cancers, such as neuroblastoma, breast cancer, and liver cancer.^26^, ^28^, ^43^, ^44^ We detected high expression levels of JMJD6 in RCC compared to normal tissues and investigated whether p300 directly binds to the promoter region and mediates the transcriptional activation of JMJD6. The p300/CBP complex is a histone acetyltransferase that promotes the establishment of H3K27ac, and p300 can activate transcription through direct interaction with RNA polymerase II. Considering that we did not detect altered expression of CBP, higher p300 levels accounted for, at least partly, the elevated JMJD6 in RCC. Whether other mechanisms, such as N6‐methyladenosine (m^6^A) modifications or ubiquitin‐mediated regulation, mediate aberrant JMJD6 levels remains unclear. The joint analysis of RNA‐seq and ChIP‐seq revealed a cluster of JMJD6 signatures that may be used as the basis for molecular classification. We selected and found several classical kidney cancer pathways, including blood vessel development, β‐catenin signaling, and cell cycle pathways.

As genomic regulatory elements, enhancers have been well studied and known to perform vital roles in controlling tissue‐specific gene expression regulation. Genomic epigenetic marks such as H3K27ac or H3K4me1/2 or coactivators (MED1, BRD4, MED1/12, and c‐Myc) are all essential enhancer indicators. In line with previous conclusions, ChIP‐seq data suggested that JMJD6 mainly binds to intragenic regions, not TSS loci, and alters enhancer profiles in RCC. As JMJD6 can interact with BRD4, we found that JMJD6 and BRD4 co‐occupy at an adjacent VEGFA region to act as a putative SE, with an accompanying H3K27ac mark. Previous studies have demonstrated that JMJD6 can activate downstream targets of E2F2, N‐Myc, and c‐Myc in neuroblastoma, indicating a role for JMJD6 as master regulator of SEs.^45^, ^46^ Recent strategies used to suppress aberrant oncogene activation by abrogating relative transcriptional SEs have been among the most promising directions, and their effects have been confirmed effectively with the BRD4 inhibitor, HDAC inhibitor, and THZ1. We treated RCC cells with JQ1 and observed a significant reduction in SE‐induced VEGFA expression. However, JQ1 and other SE inhibitors interrupt a wide range of targets across the genome and have induced a series of side effects in previous studies.^47^

Considering the vital role of JMJD6 in cancer treatment, researchers have investigated only one highly selective inhibitor, SKLB325, developed on the basis of the jmjC domain crystal structure. Accordingly, we derived SKLB325 using available protocols and determined its clinical utility in RCC organoids and PDX models. Interestingly, we further observed that JMJD6 status influenced sunitinib efficacy and combination of JMJD6 and sunitinib had synergistic effects in vitro and in vivo. As previously reported, VEGFA is one identity signature in RCC angiogenesis, participating in tumor proliferation and distal metastasis.^48^, ^49^ Encouragingly, SKLB325 could significantly suppress the tumor‐derived VEGF levels in the circulation of mice and inhibit the expressions of CD34 and CD105 in renal cell orthotopic models, which were both the angiogenesis and proliferation markers. Considering the high‐throughput sequencing data, we think that JMJD6 might activate several overlapping tyrosine kinase inhibitor downstream targets, such as SRC or FGFR1. Persistent activation of a canonical VEGF signaling bypass mediated by JMJD6 might contribute to sunitinib resistance. However, owing to publication space limits, the specific underlying resistance mechanisms induced by JMJD6 have not been thoroughly clarified. Given that JMJD6 regulating a relatively wide spectrum of targets such as BRD4, the toxicity and optimal doses of SKLB325 were warranted to evaluate based on in vitro and in vivo models.^50^, ^51^ Whether SKLB325 has an effect on a small fraction of JMJD6^low^ RCC samples to exhibit synergistic efficacy with sunitinib remains to be determined. In addition, although SKLB325 and sunitinib efficiently cooperate, the dosing quantity and order of these two drugs for optimized treatment will be of great importance to evaluate in mouse models.

## 5 CONCLUSIONS

Taken together, the results obtained by integrating the GeCK screening, multiple RCC cohort, and experimental validation data enabled us to highlight JMJD6 as an independent predictive biomarker that is also a therapeutic vulnerability for RCC. P300‐activated JMJD6 may constitute SEs to drive a series of kidney cancer‐related drivers, such as VEGFA, β‐catenin, or SRC. Targeting JMJD6 by inhibitors (SKLB325) may be an alternative strategy to suppress RCC proliferation and distant metastases. How to combine SKLB325 with traditional tyrosine kinase inhibitor drugs, such as sunitinib, to achieve optimal therapeutic benefits will be important to determine in subsequent researches.

## AUTHOR CONTRIBUTIONS

Chuanjie Zhang, Danfeng Xu, Xiaoqun Yang, and Lu Chen designed the research. Chuanjie Zhang, Xuan Lu, and Jingyi Huang conducted the experiments. Chuanjie Zhang, Xuan Lu, Jingyi Huang, and Hongchao He collected and analyzed the data. Jingyi Huang, Siwei Xing, Xiaohao Ruan, Yang Xu, Xiaoqun Yang, Hongchao He, and Haofei Wang collected the samples. Xuan Lu and Li Chen mainly conducted the pharmacology experiment of SKLB325. Chuanjie Zhang wrote and revised the manuscript. All authors read and approved the final manuscript.

## CONFLICT OF INTEREST

The authors declare no conflict of interest.

## Supporting information

Figure S1. Clinical correlation analysis between JMJD6 and clinical risk factors. (A) In the TCGA‐KIRC cohort, higher JMJD6 expression levels were positively associated with higher T stages, N stages, M status, advanced tumor grades and pathological stages.

Figure S2. JMJD6 promoted RCC progression and activated pivotal oncogenes. (A) The stable GFP‐tagged JMJD6‐overexpressing and JMJD6‐knockout RCC cell line (Caki‐1) were confirmed via western‐blotting. (B) The migration and invasion assays of 786‐O and Caki‐1 cells were conducted in control and JMJD6‐overexpressing groups, along with the statistical analysis (right panel). (C‐D) Flow cytometry analysis was conducted to detect the cytostatic effect and the occurrence of apoptosis upon JMJD6 deficiency. (E) C646, a histone acetyltransferase inhibitor targeting p300, was selected to suppress the JMJD6 mRNA levels in Caki‐1 cells in a time‐ and dose‐dependent manner. (F) Screening of enrichment of H3K27ac peaks at the promoter region of CHD6 from the data of the UCSC genome bioinformatics site (http://genome.ucsc.edu/). (G‐H) Detection of mRNA and protein levels of CHD6 upon the treatment of 40μM C646. (I‐J) Detection of mRNA and protein levels of CHD6 upon the knockdown of p300. (K) The illustration of JMJD6‐binding peaks along with H3K27ac peaks in representative targets, like FGFR1 and MAPK4.

Figure S3. Identification of essential putative JMJD6 signature. (A) By comparing the differential enhancer profiles between JMJD6 intact and deficiency cells, we further identified putative JMJD6‐regulated SEs at the indicated regions, like β‐catenin and SRC.

Figure S4. SKLB325 could suppress RCC proliferation. (A) SKLB325 was synthesized according to the instructions and the chemical structure was confirmed via Nuclear Magnetic Resonance Spectroscopy (NMRS). (B) Detection of IC50 of SKLB325 in three independent RCC cell lines. (C) SKLB525 could significantly suppress the distal lung metastases, as indicated by the statistical analysis of clones numbers (right panel). (D) Overexpressed JMJD6 significantly attenuated sunitinib efficacy compared with that observed in the control group, while JMJD6 depletion sensitized RCC cells to sunitinib. OE: over‐expression; KO: knockout; NC: WT control. (E) The CD105 expression levels were determine via IHC and corresponding statistical graph in four groups related to Figure 7I.

Supporting information

Supporting information

Supporting information

Supporting information

Supporting information

Supporting information

## Data Availability

The data used or analyzed during this study are included in this article and available from the corresponding author upon reasonable request.
