# Supplementary figures and images for: Epigenome screening highlights that JMJD6 confers an epigenetic vulnerability and mediates sunitinib sensitivity in renal cell carcinoma
Source: Clin Transl Med. 2021 Feb 14;11(2):e328. doi: 10.1002/ctm2.328 (PMC7882098; doi:10.1002/ctm2.328)

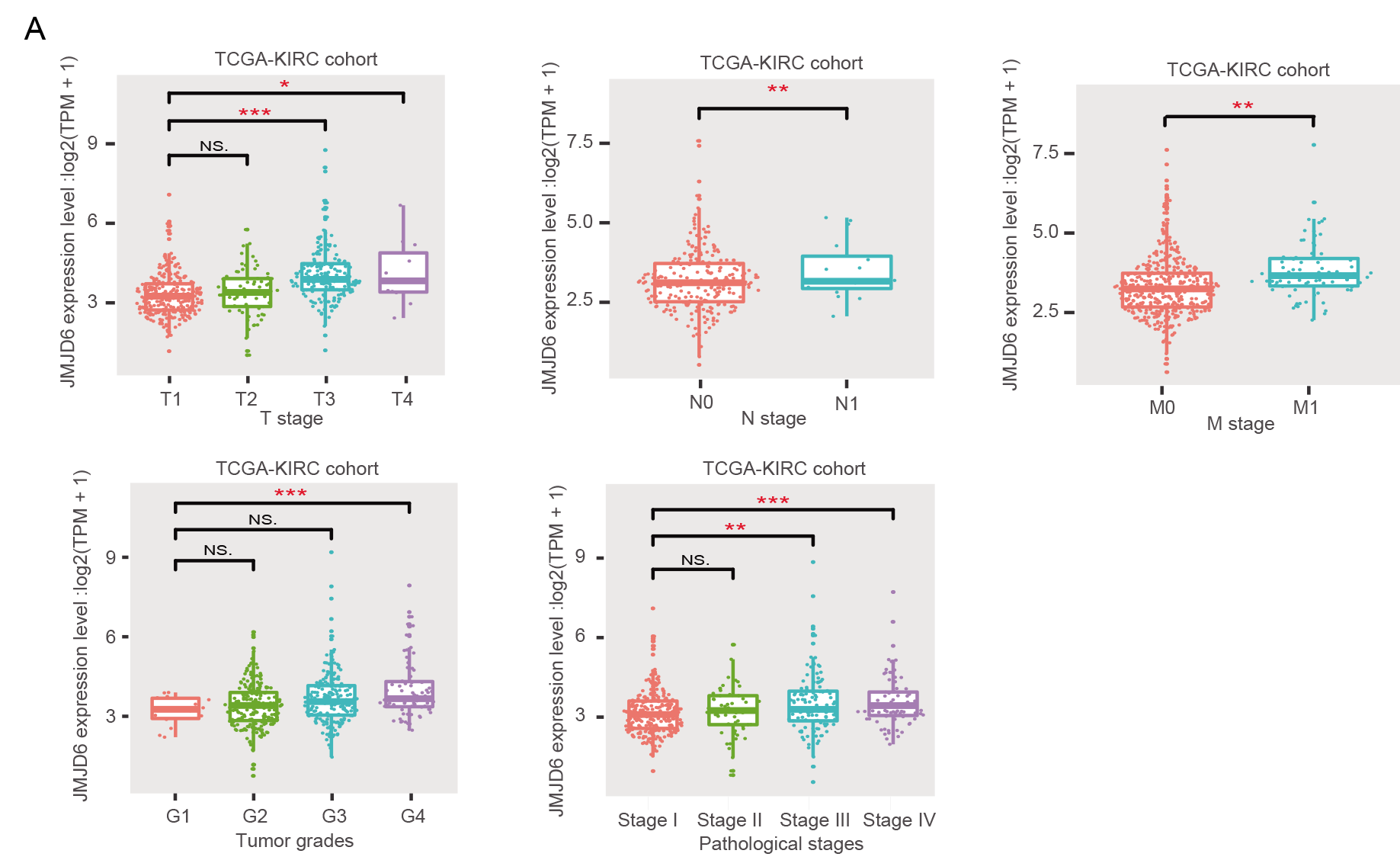

Supplement: Supplementary file 1 — Figure S1. Clinical correlation analysis between JMJD6 and clinical risk factors. (A) In the TCGA‐KIRC cohort, higher JMJD6 expression levels were positively associated with higher T stages, N stages, M status, advanced tumor grades and pathological stages. [file CTM2-11-e328-s001.tif]

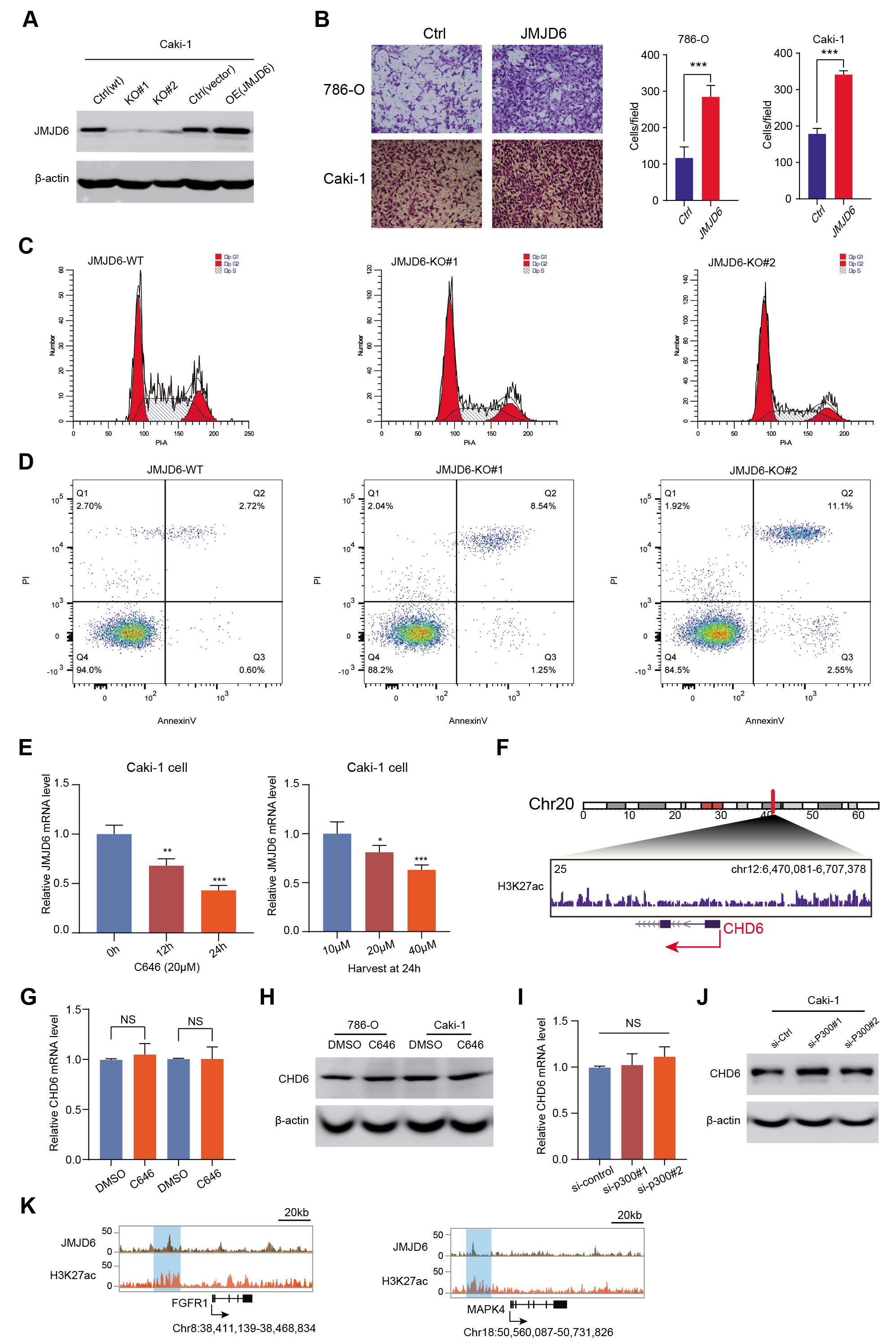

Supplement: Supplementary file 2 — Figure S2. JMJD6 promoted RCC progression and activated pivotal oncogenes. (A) The stable GFP‐tagged JMJD6‐overexpressing and JMJD6‐knockout RCC cell line (Caki‐1) were confirmed via western‐blotting. (B) The migration and invasion assays of 786‐O and Caki‐1 cells were conducted in control and JMJD6‐overexpressing groups, along with the statistical analysis (right panel). (C‐D) Flow cytometry analysis was conducted to detect the cytostatic effect and the occurrence of apoptosis upon JMJD6 deficiency. (E) C646, a histone acetyltransferase inhibitor targeting p300, was selected to suppress the JMJD6 mRNA levels in Caki‐1 cells in a time‐ and dose‐dependent manner. (F) Screening of enrichment of H3K27ac peaks at the promoter region of CHD6 from the data of the UCSC genome bioinformatics site (http://genome.ucsc.edu/). (G‐H) Detection of mRNA and protein levels of CHD6 upon the treatment of 40μM C646. (I‐J) Detection of mRNA and protein levels of CHD6 upon the knockdown of p300. (K) The illustration of JMJD6‐binding peaks along with H3K27ac peaks in representative targets, like FGFR1 and MAPK4. [file CTM2-11-e328-s002.tif]

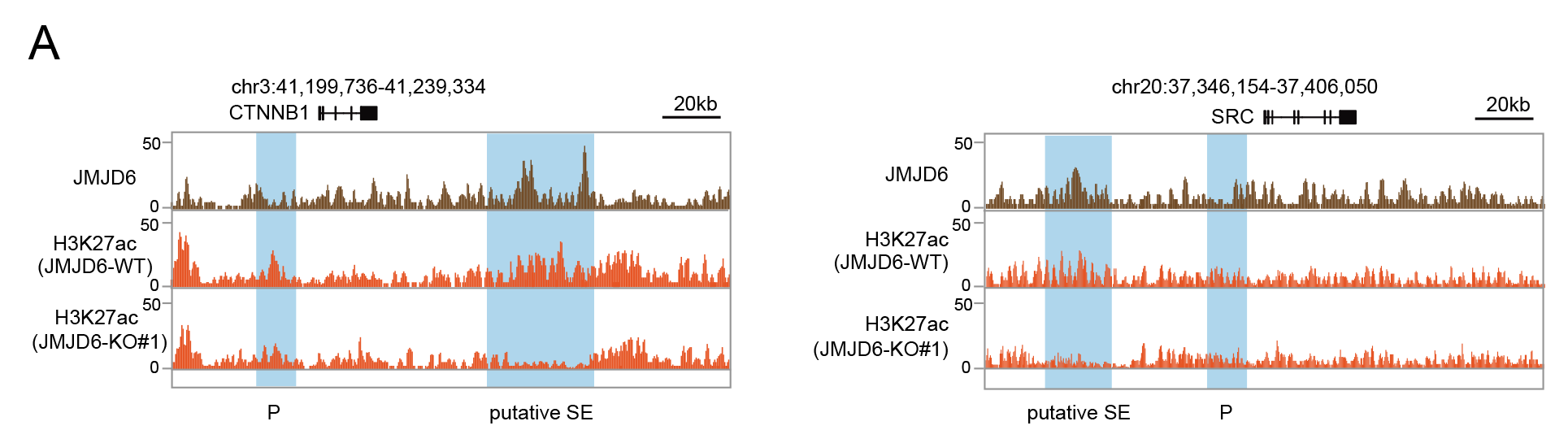

Supplement: Supplementary file 3 — Figure S3. Identification of essential putative JMJD6 signature. (A) By comparing the differential enhancer profiles between JMJD6 intact and deficiency cells, we further identified putative JMJD6‐regulated SEs at the indicated regions, like β‐catenin and SRC. [file CTM2-11-e328-s003.tif]

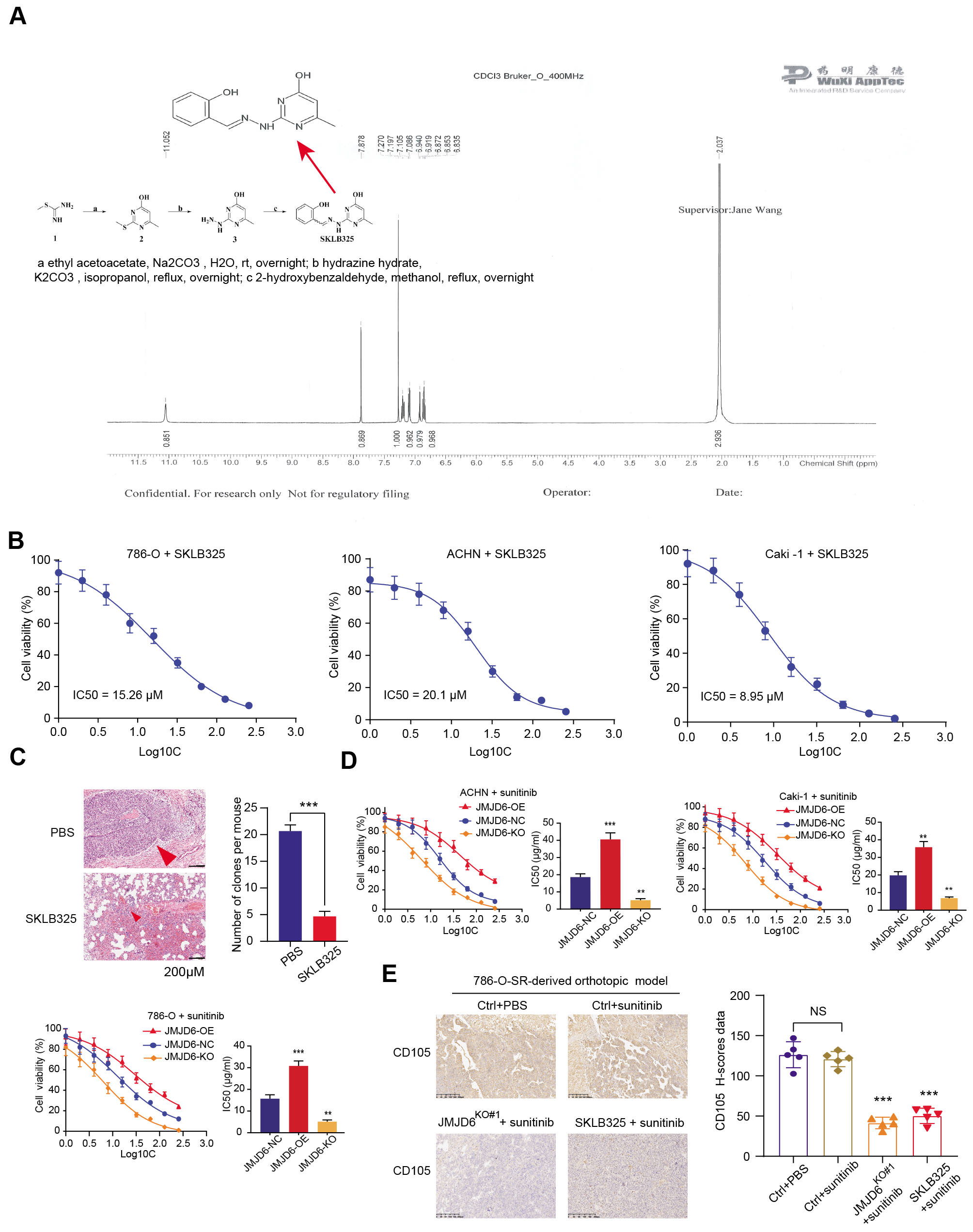

Supplement: Supplementary file 4 — Figure S4. SKLB325 could suppress RCC proliferation. (A) SKLB325 was synthesized according to the instructions and the chemical structure was confirmed via Nuclear Magnetic Resonance Spectroscopy (NMRS). (B) Detection of IC50 of SKLB325 in three independent RCC cell lines. (C) SKLB525 could significantly suppress the distal lung metastases, as indicated by the statistical analysis of clones numbers (right panel). (D) Overexpressed JMJD6 significantly attenuated sunitinib efficacy compared with that observed in the control group, while JMJD6 depletion sensitized RCC cells to sunitinib. OE: over‐expression; KO: knockout; NC: WT control. (E) The CD105 expression levels were determine via IHC and corresponding statistical graph in four groups related to Figure 7I. [file CTM2-11-e328-s004.tif]
