# Supplementary material for: Epigenome screening highlights that JMJD6 confers an epigenetic vulnerability and mediates sunitinib sensitivity in renal cell carcinoma
Source: Clin Transl Med. 2021 Feb 14;11(2):e328. doi: 10.1002/ctm2.328 (PMC7882098; doi:10.1002/ctm2.328)
Supplement: Supplementary file 5 — Supporting information [file CTM2-11-e328-s005.docx]

**Table S1. Primers, sequences of shRNAs and siRNAs, antibody and chemicals.**

| **Primers for RT-qPCR with cell lines samples** | | | | | |
| --- | --- | --- | --- | --- | --- |
| Gene name | F: 5'-3' | | | | R: 5'-3' |
| JMJD6 | CCAACTTCCCTGTGGTATGGCA | | | | TCCTGAAGGTCAACCGAGTCTG |
| VEGFA | TTGCCTTGCTGCTCTACCTCCA | | | | GATGGCAGTAGCTGCGCTGATA |
| VEGFB | AAGGACAGTGCTGTGAAGCCAG | | | | TGGAGTGGGATGGGTGATGTCA |
| SRC | AAGGTGGAGCACTACCGCATCA | | | | AGTCCATCTGCGTCTGAGGTGT |
| CDC42 | TGACAGATTACGACCGCTGAGTT | | | | GGAGTCTTTGGACAGTGGTGAG |
| CTNNB1 | CACAAGCAGAGTGCTGAAGGTG | | | | GATTCCTGAGAGTCCAAAGACAG |
| CCND1 | TCTACACCGACAACTCCATCCG | | | | TCTGGCATTTTGGAGAGGAAGTG |
| MMP9 | GCCACTACTGTGCCTTTGAGTC | | | | CCCTCAGAGAATCGCCAGTACT |
| CDK6 | GGATAAAGTTCCAGAGCCTGGAG | | | | GCGATGCACTACTCGGTGTGAA |
| CDKN2D | GTGCATCCCGACGCCCTCAAC | | | | TGGCACCTTGCTTCAGCAGCTC |
| HDGF | CCAAAGACCTCTTCCCTTACGAG | | | | TGGTTCAGGCTCTTCCACACAG |
| CBP | AGTAACGGCACAGCCTCTCAGT | | | | CCTGTCGATACAGTGCTTCTAGG |
| P300 | GATGACCCTTCCCAGCCTCAAA | | | | GCCAGATGATCTCATGGTGAAGG |
| **Sequences of sgRNAs for indicated genes** | | | | | |
| JMJD6#1 | | ACCGTCTTGTGCCATACCAC | | |  |
| JMJD6#2 | | ATCAAAGTGACCCGAGACGA | | |  |
| **­­­­Sequences of shRNAs for indicated genes** | | | | | |
| Gene name | Sequence | | | | |
| JMJD6#1 | CCGGATGGACTCTGGAGCGCCTAAACTCGAGTTTAGGCGCTCCAGAGTCCATTTTTTG | | | | |
| JMJD6#2 | CCGGGCGGTATGAAAGACCTTACAACTCGAGTTGTAAGGTCTTTCATACCGCTTTTTG | | | | |
| VEGFA#1 | GCAGATTATGCGGATCAAACC | | | | |
| VEGFA#2 | GCCAGCACATAGGAGAGATGA | | | | |
| CTNNB1#1 | GGTTAATAAGGCTGCAGTTAT | | | | |
| CTNNB1#2 | GCTTATGGCAACCAAGAAAGC | | | | |
| **Sequences of siRNAs for indicated genes** | | | | | |
| Gene name | Sequence | | | | |
| AURKA | GCATTGGAGTGCTTTGCTA | | | |  |
| AURKB | GGAGGAGGATCTACTTGAT | | | |  |
| CAPRIN2 | GCCTGCAGTTTCCTTAGAA | | | |  |
| CBX8 | GCATACGGAAAGGACGCAT | | | |  |
| EPAS1 | GCCACAGCATGGACATGAA | | | |  |
| HDAC10 | GCAGCTCTAAGCAAAGCTG | | | |  |
| KAT2A | GCCAAGGACCAATGCAGTA | | | |  |
| JMJD6 | GGAGAGCACTCGAGATGAT | | | |  |
| MYBL2 | GCCACTGAGCGCAAATGTA | | | |  |
| PHF19 | GGGTCCTATGGAAGGACAT | | | |  |
| PLK1 | GCAAATGTACCCAATGATA | | | |  |
| P300 | GCAGCTCAACCATCCACTA | | | |  |
| SIRT7 | GCGAAGGTCAAGAACAACT | | | |  |
| **Antibody&Chemicals** | | | | |  |
| **Name** | | | **Species** | **Cat. No** | **Source** |
| JMJD6 | | | Mouse | ab64575 | abcam |
| P300 | | | Mouse | ab275378 | abcam |
| H3K27ac | | | Mouse | ab4729 | abcam |
| BRD4 | | | Mouse | ab128874 | abcam |
| VEGFA | | | Mouse | 66828-1-Ig | Proteintech |
| VEGFR-2 | | | Mouse | ab134191 | abcam |
| CTNNB1 | | | Mouse | ab32572 | abcam |
| Actin | | | Mouse | ab8226 | abcam |
| puromycin | | |  | S7417 | Selleckchem |
| **Organoids culture** | | | | | |
| **Additive** | | | **Source** | **Cat. No.** | **Concentration** |
| EGF | | | PeproTech | AF-100-15 | 50 ng/ml |
| Noggin | | | PeproTech | 250-38 | 100 ng/ml |
| R-Spondin 1 | | | PeproTech | 120-38 | 500 ng/ml |
| Gastrin | | | Sigma-Aldrich | G9145 | 10 nM |
| FGF-10 | | | PeproTech | 100-26 | 10 ng/ml |
| FGF-basic | | | PeproTech | 100-18B | 10 ng/ml |
| Wnt-3A | | | R&D Systems | 5036-WN | 100 ng/ml |
| Prostaglandin E2 | | | Tocris Bioscience | 2296 | 1 µM |
| Y-27632 | | | Sigma-Aldrich | Y0503 | 10 µM |
| Nicotinamide | | | Sigma-Aldrich | N0636 | 4 mM |
| A83-01 | | | Tocris Bioscience | 2939 | 0.5 µM |
| SB202190 | | | Sigma-Aldrich | S7067 | 5 µM |
| HGF* | | | PeproTech | 100-39 | 20 ng/ml |
