# Supplementary material for: Epigenome screening highlights that JMJD6 confers an epigenetic vulnerability and mediates sunitinib sensitivity in renal cell carcinoma
Source: Clin Transl Med. 2021 Feb 14;11(2):e328. doi: 10.1002/ctm2.328 (PMC7882098; doi:10.1002/ctm2.328)
Supplement: Supplementary file 7 — Supporting information [file CTM2-11-e328-s007.docx]

Table S3. Clinical features of public RCC patients included in this study.

| \| **Variables** \| \| **TCGA** \| **ICGC** \| \| --- \| --- \| --- \| --- \| \|  \| \| (N = 537) \| (N = 91) \| \| **Age (Mean ± SD)** \| \| 60.59 ± 12.14 \| 60.47 ± 9.97 \| \| **Follow-up (y)** \| \| 3.12 ± 2.23 \| 4.14 ± 1.73 \| \| **Status** \|  \|  \|  \| \| Alive \|  \| 367 (68.34) \| 61 (67.03) \| \| Dead \|  \| 170 (31.66) \| 30 (32.97) \| \| **Gender** \|  \|  \|  \| \| Male \|  \| 346 (64.43) \| 52 (57.14) \| \| Female \|  \| 191 (35.57) \| 39 (42.86) \| \| **AJCC-T** \|  \|  \|  \| \| T1 \|  \| 275 (51.21) \| 54 (59.34) \| \| T2 \|  \| 69 (12.85) \| 13 (14.28) \| \| T3 \|  \| 182 (33.89) \| 22 (24.18) \| \| T4 \|  \| 11 (2.05) \| 2 (2.20) \| \| **AJCC-N** \|  \|  \|  \| \| N0 \|  \| 240 (44.69) \| 79 (86.81) \| \| N1 \|  \| 17 (3.17) \| 2 (2.20) \| \| Unknow \|  \| 280 (52.14) \| 10 (10.99) \| \| **AJCC-M** \|  \|  \|  \| \| M0 \|  \| 426 (79.33) \| 81 (89.01) \| \| M1 \|  \| 79 (14.71) \| 9 (9.89) \| \| Unknow \|  \| 32 (5.96) \| 1 (1.10) \| \| **Pathological stage** \|  \|  \|  \| \| I \|  \| 269 (50.09) \| - \| \| II \|  \| 57 (10.61) \| - \| \| III \|  \| 125 (23.28) \| - \| \| IV \|  \| 83 (15.46) \| - \| \| Unknow \|  \| 3 (0.56) \| - \| \| **Grade** \|  \|  \|  \| \| G1 \|  \| 14 (2.61) \| - \| \| G2 \|  \| 230 (42.83) \| - \| \| G3 \|  \| 207 (38.54) \| - \| \| G4 \|  \| 78(14.53) \| - \| \| Unknow \|  \| 8(1.49) \| - \| |
| --- | --- | --- | --- | --- | --- | --- | --- | --- | --- | --- | --- | --- | --- | --- | --- | --- | --- | --- | --- | --- | --- | --- | --- | --- | --- | --- | --- | --- | --- | --- | --- | --- | --- | --- | --- | --- | --- | --- | --- | --- | --- | --- | --- | --- | --- | --- | --- | --- | --- | --- | --- | --- | --- | --- | --- | --- | --- | --- | --- | --- | --- | --- | --- | --- | --- | --- | --- | --- | --- | --- | --- | --- | --- | --- | --- | --- | --- | --- | --- | --- | --- | --- | --- | --- | --- | --- | --- | --- | --- | --- | --- | --- | --- | --- | --- | --- | --- | --- | --- | --- | --- | --- | --- | --- | --- | --- | --- | --- | --- | --- | --- | --- | --- | --- | --- | --- | --- | --- | --- | --- | --- | --- | --- | --- | --- | --- | --- | --- | --- | --- | --- | --- | --- | --- | --- | --- | --- | --- | --- | --- |

Data are shown as n (%).

**Abbreviations:** TCGA, The Cancer Genome Atlas; ICGC, International Cancer Genome Consortium; AJCC, American Joint Committee on Cancer.
